# Therapeutic targeting of the AMPK-Has1 complex formation ameliorates metabolic dysfunction-associated steatohepatitis in mice

**DOI:** 10.7150/thno.120527

**Published:** 2026-01-22

**Authors:** Xunzhe Yin, Wenjing Zhao, Li Yang, Chang Li, Xiangyu Guo, Lihao Lin, Zuojia Liu, Jin Wang

**Affiliations:** 1 Center for Theoretical Interdisciplinary Sciences, Wenzhou Institute, University of Chinese Academy of Sciences, Wenzhou, Zhejiang 325001, China.; 2 State Key Laboratory of Electroanalytical Chemistry, Changchun Institute of Applied Chemistry, Chinese Academy of Sciences, Changchun, Jilin 130022, China.; 3 Department of Chemistry and of Physics and Astronomy, Stony Brook University, Stony Brook, NY 11794-3400, USA.; 4 Hepatobiliary Hospital of Jilin Province, Changchun, Jilin 130062, China.

**Keywords:** MASH, elemicin, Has1, AMPK, multi-omics

## Abstract

**Rationale:** Metabolic dysfunction-associated steatohepatitis (MASH) is a severe liver disease with limited therapeutic options. This study aimed to investigate the protective effects of elemicin (Ele) against MASH and its underlying mechanisms, focusing on the interaction between AMP-activated protein kinase (AMPK) and hyaluronan synthase 1 (Has1).

**Methods:** HFHC diet-induced MASH mouse models and palmitic acid/oleic acid (PO)-treated primary hepatocytes were used. Transcriptomic and lipidomic analyses, immunohistochemistry, western blotting, and molecular docking were employed to assess gene expression, lipid metabolism, inflammation, and fibrosis. Interactions between Ele, AMPK, and Has1 were validated *via* SPR, Co-IP, and CETSA.

**Results:** Ele significantly ameliorated hepatic steatosis, inflammation, and fibrosis in MASH mice. Systematic profiling of transcriptomic and lipidomic landscapes reveals that Has1-mediated lipid metabolism is strongly correlated with MASH severity in dietary mouse models. Using loss-of-function studies, liver-specific inhibition of Has1 ameliorates hepatic steatosis, inflammation and fibrosis *in vivo* and *in vitro*. The anti-MASH effects of Ele are largely dependent on interrupting the formation of AMPK/Has1 complex. Furthermore, Ele normalized hepatic phospholipid profiles, particularly increasing phosphatidylethanolamine to improve mitochondrial function.

**Conclusions:** Ele protects against MASH by interrupting AMPK/Has1 interaction, regulating lipid metabolism, and restoring mitochondrial function. Collectively, these findings highlight Ele as a potential therapeutic agent and Has1 as a novel target for MASH treatment.

## Introduction

Metabolic dysfunction-associated steatotic liver disease (MASLD), the condition formerly termed nonalcoholic fatty liver disease (NAFLD), has recently gained recognition as a major contributor to chronic liver pathology across the globe 1,2. MASLD represents a disease continuum consisting of metabolic-associated steatotic liver (MASL), MASH, fibrosis, cirrhosis, and hepatocellular carcinoma 3. While MASL is considered a reversible and benign disease, it is noteworthy that a subset of MASL patients may advance to MASH and subsequently to irreversible advanced stages 2. Until recently, approved drugs for MASH therapy remained elusive in clinical practice, with resmetirom being the first to receive approval 4. Therefore, the identification of novel therapeutic targets for MASH remains a critical need.

AMP-activated protein kinase (AMPK) is essential for coordinating changes in hepatic metabolism needed to maintain energy homeostasis 5. An advantage of activating AMPK in the liver is that AMPK is capable of inhibiting *de novo* lipogenesis (DNL) and promotes fatty acid oxidation (FAO) *via* phosphorylation and inactivation of acetyl-CoA carboxylase (ACC) 6. Given its pivotal role, AMPK has become a promising target for treating diseases arising from disrupted energy metabolism. Hyaluronan (HA) is a glycosaminoglycan primarily synthesized by hyaluronan synthases (Has), serving diverse functions in tissue injury and repair, including regulation of inflammation, tissue damage, and fibrosis 7,8. Serum HA levels are markedly elevated in metabolic and fibrotic liver disorders, including MASLD, MASH, and diabetes, and have been proposed as a useful non-invasive indicator for disease progression and hepatic injury 9. More importantly, accumulating evidence suggests that Has1, as one of the hyaluronan synthases, plays a crucial role in modulating inflammatory processes and metabolic homeostasis in conditions characterized by altered glucose metabolism. In addition, dysregulations of HA levels and Has1 expression in MASH accompanied by fibrosis can disrupt the functional and structural homeostasis of hepatic adipocytes, exacerbating the progression of MASH. Interestingly, it has been reported that AMPK can regulate the lipid microenvironment surrounding the Has1 enzyme, indirectly modulating its activity or stability 8. This occurs because AMPK activation dramatically inhibits enzymatic activity, thereby reducing HA secretion 10. To date, there remain limited reports on the association between Has1 and AMPK in MASH, warranting to be further explored.

MASH is a complex disease involving dysregulation of multiple interconnected biological pathways as described above. For instance, mitochondria are indispensable for maintaining hepatic metabolic homeostasis. In patients with MASH, mitochondrial impairment is frequently observed and contributes to the pathological progression from simple steatosis to steatohepatitis 11. Evidence from clinical studies further reveals that alterations in mitochondrial lipid composition influence hepatic performance 12,13. Notably, phospholipids modulate mitochondrial behavior, including its morphology, respiratory efficiency, and protein quality control 13. In high-fat/high-cholesterol (HFHC) diet-induced murine models, levels of mitochondrial phosphatidylethanolamine (PE), phosphatidylcholine (PC), phosphatidylserines (PS), phosphatidate (PA), and phosphatidylinositols (PI) show significant changes, which further disturb mitochondrial ATP level in hepatocytes 14. Although mitochondrial phospholipids are believed to participate in hepatic metabolic regulation, their specific involvement in MASH pathogenesis has yet to be clarified. Lipidomics provides a powerful approach for profiling alterations in endogenous lipid metabolism and is designed to precisely identify lipid composition and biomarkers at the molecular level to investigate the pathogenesis of lipid metabolism-related diseases.

Most recently, we employed a distinctive library of bioactive natural compounds to identify potential therapeutics for MASH using primary mouse hepatocytes treated with palmitic acid/oleic acid (PO). A typical natural alkenylbenzene and low molecular weight bioactive compound, 3,4,5-trimethoxyallylbenzene (also known as elemicin, Ele), was picked for exhibiting remarkable anti-MASH effects. Ele occurs abundantly in numerous medicinal herbs and constitutes an aromatic ingredient in foods such as baked goods, meats, soft drinks, and ice cream. It has recently gained research interest due to a promising pharmacological profile that encompasses antimicrobial, antioxidant, and antiviral properties 15,16. The integrative transcriptomic and lipidomic analysis unveiled elevated hepatic Has1 expression and metabolic disorders in HFHC diet-induced MASH mouse models, while Ele effectively suppressed MASH progression by weakening the AMPK-Has1 complex formation. Therefore, the current findings reveal a previously unknown anti-MASH mechanism and identify a promising therapeutic compound for MASH.

## Material and Methods

### Materials and reagents

Elemicin (Ele, purity ≥ 98%, CAS: 487-11-6) was obtained from Selleck (Houston, USA). Cell culture media, including Dulbecco's modified Eagle medium (DMEM) and Roswell Park Memorial Institute (RPMI)-1640 medium, were obtained from Gibco (New York, USA), while fetal bovine serum (FBS), streptomycin, and penicillin were sourced from Biological Industries (Beit Haemek, Israel). Bovine serum albumin (BSA), dimethyl sulfoxide (DMSO), oleic acid (OA), and palmitic acid (PA) were purchased from Sigma-Aldrich (St. Louis, USA). Assay kits for triglyceride (TG), total cholesterol (TC), interleukin-1β (IL-1β), low-density lipoprotein cholesterol (LDL-C), aspartate transaminase (AST), creatinine (Cr), blood urea nitrogen (BUN), interleukin-18 (IL-18), alanine transaminase (ALT), and dehydrogenase (LDH) were supplied from Jiancheng Biological Engineering Research Institute (Nanjing, China). Primary antibody against NLRP3, TGFβ, and Col1a1 was obtained from Abmart (Shanghai, China). Primary antibodies against p-AMPK, AMPK, p-ACC, ACC, SREBP-1c, FASN, PPARα, CPT-1A, SCD1, ASC, and β-actin were provided by Abcam (Cambridge, UK). Primary antibody against Has1 and Pisd supplied by Boster (Wuhan, China). Anti-mouse or anti-rabbit horseradish peroxidase (HRP)-conjugated secondary antibodies were obtained from ZSGB-Bio (Beijing, China). The chemiluminescent HRP substrate was provided from Millipore (Billerica, USA), and the recombinant Has1 protein was provided by Feiyue Biotechnology (Wuhan, China). Cell Counting Kit-8 (CCK8), BCA protein assay kit, and protein A/G magnetic agarose beads were purchased from Beyotime Biotechnology (Shanghai, China). The UNIQ-10 column trizol total RNA isolation kit was supplied from Sangon Biotech (Shanghai, China). Stains Oil Red O (ORO) and Nile Red were supplied by Solarbio (Beijing, China). All primers were synthesized by Comate Bioscience (Changchun, China). The RNA reverse transcription kit and SYBR qPCR Master Mix were obtained from Promega (Madison, USA). SiRNAs and riboFECT CP transfection reagent were supplied by RiboBio (Guangzhou, China). Mito stress and glycolysis stress test kits were provided by Agilent Technologies (Santa Clara, USA). For all experiments, the final concentrations of DMSO were maintained below 0.1% (v/v).

### Animals and treatments

Eight-week-old male C57BL/6J mice were obtained from Vital River Laboratory Animal Technology Co., Ltd (Beijing, China), were housed under standard conditions (23 ± 2 °C, 12 h light/dark cycle) with unrestricted access to food and water. To induce the MASH models, mice were fed an HFHC diet containing 42% fat, 22% fructose, and 2% cholesterol (Vital River, Beijing, China) for 16 weeks. Control mice received a normal chow diet. During the experimental phase, treatment groups were administered either Ele (100 or 200 mg/kg) or 4-methylumbelliferone (4-MU, 200 mg/kg; Selleck, USA) *via* intragastric gavage every other day for 8 weeks, while control groups received parallel handling. The 16-week HFHC diet model was chosen because it closely mimics the gradual transition from obesity and metabolic dysfunction to steatohepatitis with fibrosis, thereby providing a clinically relevant context for exploring the contribution of metabolic disorders to MASH pathogenesis. Owing to its reproducibility and translational value, the HFHC model is widely regarded as a benchmark in preclinical pharmacological studies. All experimental procedures were conducted in accordance with the Guide for the Care and Use of Laboratory Animals published by the National Academy of Sciences and the National Institutes of Health. Apart from exhibiting obesity, the animals remained in overall good health throughout the study.

### Human liver samples and blood samples

Human liver specimens were collected from adult patients with MASH who underwent liver transplantation or biopsy. Non-steatotic liver tissues were obtained from the normal donor livers. In total, the study included six non-steatotic and seven MASH liver samples. Exclusion criteria for liver samples comprised a history of excessive alcohol consumption (defined as > 140 g/week for males or > 70 g/week for females), drug abuse, or viral infections such as HBV and HCV. Steatosis and steatohepatitis were evaluated independently by a pair of pathologists, following the standardized histological criteria established by the MASH Clinical Research Network. A diagnosis of MASH was assigned to samples exhibiting a NAFLD activity score (NAS) ≥ 5, or a NAS of 3-4 accompanied by fibrosis, whereas samples with a NAS of 0 were defined as normal. To ensure specificity, cases were excluded if they involved HBV/HCV infection, daily alcohol intake exceeding 20 g, or other known causes of chronic liver disease. Following an 8-hour overnight fast, peripheral venous blood samples were obtained from the ante cubital vein. All procedures were consistent with the principles outlined in the Declaration of Helsinki.

### Cell culture and treatment

Primary hepatocytes are regarded as the principal cellular component of liver architecture and play a crucial role in metabolic disorders. Mouse primary hepatocytes were isolated from the livers of 6- to 8-week-old C57BL/6J male mice by a two-step collagenase perfusion process.

Briefly, mice were anesthetized using 1.2% tribromoethyl alcohol (Sigma-Aldrich, St. Louis, USA). Subsequently, the liver was perfused *via* the portal vein with liver perfusion medium (Thermo Fisher Scientfic, Waltham, USA), followed by liver digestion medium (Thermo Fisher Scientfic, Waltham, USA) at 37 °C for approximately 20 min. The digested liver was then excised, minced, and passed through a 70 µm cell strainer. Hepatocytes were isolated by two rounds of centrifugations at 50 g for 2 min each. The resulting primary hepatocytes were maintained in DMEM containing with 10% FBS and 1% penicillin-streptomycin for subsequent experiments. The human hepatocyte L02 cell line was kindly provided by Dr. Shiting Yu (Changchun University of Chinese Medicine, Changchun, China) and maintained in DMEM containing with 10% FBS and 1% penicillin-streptomycin. All cell cultures were maintained under standard conditions (37 °C, 5% CO_2_) in a humidified incubator and were confirmed to be free of mycoplasma contamination.

### Histopathological analysis

For histological analysis, vital organs (lung, heart, spleen, liver, kidney) were collected from HFHC-fed mice, fixed in formalin, and embedded in paraffin. Sections were prepared and routinely stained with H&E. To specifically characterize liver pathology, additional staining with ORO solution, Masson, and Picrosirius Red (PSR) was performed on liver tissue according to the kit instructions. The NAS was subsequently assessed based on the histopathological examination of liver sections, evaluating the extent of steatosis, lobular inflammation, and hepatocyte ballooning.

### Measurement of serum and hepatic biochemical index

Cellular levels of TG and TC were determined by enzymatic colorimetric kits with the employment of a Tecan Infinite M200 Pro microplate reader (Zurich, Switzerland). Serum and hepatic biochemical parameters were determined following the protocols provided by the assay kit manufacturer. The levels of AST, ALT, TC, TG, LDL-C, Cr, and BUN were subsequently determined.

### RNA-sequencing

Total RNA was extracted from mouse liver tissues and RNA purity was detected by as follows (i) analyzing the integrity of RNA and detecting the presence of DNA contamination using agarose gel electrophoresis; (ii) precisely measuring RNA concentration with high accuracy using the Qubit 2.0 Fluorometer; (iii) accurately evaluating RNA integrity using an Agilent Bioanalyzer 2100 system (Santa Clara, USA). cDNA libraries were prepared to profile gene expression differences and subsequently sequenced on an Illumina Novaseq platform (San Diego, USA). This RNA-sequencing service was provided by Metware Biotechnology Inc. (Wuhan, China).

### Western blotting

Briefly, total proteins from cultured cells or mouse liver tissues were extracted using RIPA lysis buffer supplemented with protease and phosphatase inhibitors. Protein concentrations were determined using a BCA assay kit. Equal amounts of protein were separated by 10% sodium dodecyl sulfate polyacrylamide (SDS-PAGE) gel and subsequently electrotransferred onto polyvinylidene difluoride (PVDF) membranes (Millipore, Billerica, USA). Following a blocking step with 5% skim milk in TBST, the membranes were incubated overnight at 4 °C with the indicated primary antibodies, followed by HRP-conjugated secondary antibodies for 1 h at room temperature. Protein bands were visualized by a chemiluminescent HRP substrate and detected with a Bio-Imaging System (DNR, Israel). All band intensities were quantified using ImageJ180 software (Maryland, USA), with β-actin serving as a loading control.

### Cytotoxicity and ELISA assays

Cell lipotoxicity was evaluated using a CCK8 assay and LDH release assay. For the LDH assay, culture medium was collected and analyzed according to the kit protocol. For the CCK8 assay, mouse primary hepatocytes were seeded in 96-well plates at a density of 5,000 cells per well. After treatment with 50 or 100 μM Ele for the indicated durations, CCK8 reagent was added to each well, followed by incubation for 1-3 h at 37 °C. Absorbance at 450 nm was then measured to determine cell viability. Concurrently, serum levels of IL-1β and IL-18 in mice were quantified by ELISA, while hyaluronan concentrations were determined using a specific quantitative sandwich enzyme immunoassay kit (Solarbio, Beijing, China), both performed in accordance with the manufacturers' instructions.

### Real-time qPCR

Total RNA was isolated using Trizol reagent, and complementary DNA (cDNA) was synthesized with the PrimeScript RT reagent Kit with gDNA Eraser Kit (TaKaRa, Kyoto, Japan) according to the manufacturer's protocol. Quantitative real-time PCR was conducted using SYBR qPCR Master Mix (Promega, USA). Relative mRNA expression levels were calculated using the 2^-ΔΔCt^ method and normalized to β-actin expression. All primer sequences detailed in Table S1 (Supplementary Material).

### Cellular Nile Red and Oil Red O staining

Primary hepatocytes and L02 cells were treated with 1 mM PA/OA, and then the cells were fixed with 4% paraformaldehyde and subsequently rinsed with 60% isopropanol solution. Cells were stained with ORO. Mayer's hematoxylin was added. In addition, after being fixed with paraformaldehyde, primary hepatocytes and L02 cells were stained with Nile Red. After incubation, cells were mounted in with DAPI (Biosharp, Hefei, China). Cell images were acquired with a Nikon laser-scanning confocal microscope (Nikon, Tokyo Metropolis, Japan).

### Immunfluorescence staining

After overnight culture on glass-bottom dishes (NEST Biotech, China), primary hepatocytes underwent standard immunofluorescence staining. Briefly, cells were fixed with 4% paraformaldehyde and permeabilized with 0.1% Triton X-100 at room temperature. A 2-h block was applied to prevent non-specific binding before an overnight incubation with primary antibodies at 4 °C. Cells were exposed to fluorescent secondary antibodies. Imaging was performed on a Nikon confocal microscope.

### Metabolic assays

Cells were seeded into XFp microplates the day before determination. Cellular bioenergetics were assessed in real-time by measuring the oxygen consumption rate (OCR) and extracellular acidification rate (ECAR) using an Agilent Seahorse XFp Analyzer (Santa Clara, USA). Before measurement, microplates were washed, and cells were incubated in a CO₂-free incubator to equilibrate. Metabolic assays were conducted in XF assay medium (pH 7.3-7.4) supplemented with 2.5 M glucose, 0.2 M glutamine, and 0.1 M sodium pyruvate. OCR was assessed using sequential injections of 1 µM oligomycin, 1 µM FCCP, and 0.5 µM rotenone. ECAR was determined in medium containing 0.2 mM glutamine, 10 mM glucose, 10 µM oligomycin, and 50 mM 2-deoxy-D-glucose (2-DG). All data were acquired and analyzed using Agilent Wave Desktop Software (Santa Clara, USA).

### Immunohistochemistry (IHC)

Briefly, IHC of Has1 activities were performed on paraffin embedded liver sections using Has1 antibody. The sections were then dewaxed and rehydrated, followed by antigen retrieval. The sections were blocked and then incubated with primary antibody against Has1 at 4 °C overnight. After rewarming, samples were washed and then incubated with secondary antibodies. Immunohistochemical staining was visualized using diaminobenzidine, followed by hematoxylin counterstaining. Images were captured on a Thermo light microscope (Waltham, USA).

### SiRNA transfection

SiRNA and siNC oligonucleotide sequences were synthesized by Ribobio (Guangzhou, China). Cells were transfected with siRNAs targeting Has1 and AMPK using the riboFECT CP reagent, according to the manufacturer's protocol. Transfection efficiency was then determined by quantifying the corresponding protein levels *via* western blot analysis.

### Co-immunoprecipitation (Co-IP) analysis

Cells were homogenized in IP-specific lysis buffer containing inhibitors at 4 °C. The lysates were centrifuged at 10,000-14,000 × g for 10 min to collect the supernatant, which was then incubated with protein A/G magnetic agarose beads overnight at 4 °C with gentle mixing. The bead-bound complexes were subsequently immunoprecipitated with the indicated antibodies overnight at 4 °C. After washing, the immunocomplexes were eluted with SDS-PAGE loading buffer and analyzed by immunoblotting using the relevant primary and secondary antibodies.

### Molecular docking

To predict the binding interaction between Ele on Has1, the three-dimensional structure of Ele (CID: 10248) was retrieved from the PubChem database and constructed using Open Babel software. The crystal structure of mouse Has1 (Uniprot ID: Q61647) was used as input. Ele was specified as the ligand, and Has1 protein was designated as the receptor. Following preparation with AutoDockTools (The Scripps Research Institute, CA, USA), docking simulations were performed with Autodock Vina 1.1.2 (The Scripps Research Institute, CA, USA) to predict the binding pose of Ele within the identified cavity of Has1. Further, the complexes were subjected to three-dimensional and two-dimensional force analyses and visualizations using Pymol (DeLano Scientific, San Francisco, USA) and Discovery Studio (BIOVIA, San Diego, USA) software.

### Surface plasmon resonance (SPR)

The direct interaction between Ele and Has1 protein was authenticated through SPR. SPR assay was conducted on a REICHERT 4SPR system (New York, USA) equipped with a CM5 sensor chip. The Has1 protein (100 μg/mL) was immobilized onto the chip *via* amine coupling in 10 mM ethanolamine (pH 4.5). Serial concentrations of Ele prepared in running buffer (1 × PBST, 0.1% DMSO) were flowed over the chip to generate response signals. The association and dissociation phases were continuously monitored in real time and kinetics as well as affinity parameters were analyzed using TraceDrawer software (Ridgeview Instruments AB, Vänge, Sweden).

### Cellular thermal shift assay (CETSA)

The capacity of Ele to interact with and stabilize Has1 protein within intact cells was primarily assessed using CETSA. Subsequent to treatment, harvested cells were centrifuged and then resuspended. The resuspended cells were lysed on ice and subsequently centrifuged. The resulting cell suspensions were divided into multiple aliquots, placed into microtubes, and heated for 3 min at temperatures ranging from 44, 46, 48, 50, 52, 54, 56, 58, and 60 °C, with intervals of 2 °C. After rapid cooling at room temperature, precipitates were subsequently separated from the heat-treated lysates and subjected to analysis through western blotting.

### Isothermal titration calorimetry (ITC)

The binding of Ele to the Has1 protein was examined by Nano ITC (TA Instruments, New Castle, USA). The calorimetric cells were automatically filled with Has1 protein suspended in PBS solution containing 1% DMSO. Concurrently, a syringe was loaded with PBS solution (1% DMSO) containing Ele. The titrant was titrated into solution or buffer in the calorimetric cell at 200 s intervals. The experiment was conducted at 25 °C with a stirring speed of 750 rpm. The injection volume of the titrant was 2 µl. The titration of the substrate solution (titrate) with a blank solution (titrant) was conducted to establish the background signal. Raw data were processed using NanoAnalyze software (TA Instruments, New Castle, USA), and the integrated heat effects were quantified through nonlinear regression analysis employing a single-site binding model.

### Lipidomic analysis

After thawing the samples (*n* = 6), the mouse liver tissues were weighed and placed into corresponding pre-numbered centrifuge tubes. Subsequently, lipid extraction solution was added, followed by the addition of steel beads, and the mixture was homogenized. The steel beads were removed using a ball mill, and the resulting homogenate was vortexed, ultrasonic decomposed, and water was added. The mixture was vortexed again, and centrifuged at 4 °C. The upper clear liquid was aspirated into corresponding numbered centrifuge tubes, concentrated, and reconstituted with a lipid resolubilization solution. Lipidomic profiling was performed by Metware Biotechnology Inc. (Wuhan, China) using a SCIEX UPLC-MS/MS system. Lipids were qualitatively identified against the proprietary MWDB based on retention time and MS/MS fragments, and quantitatively measured* via* MRM mode on a triple quadrupole mass spectrometer. Data processing involved peak area integration and normalization using internal standards.

### Statistical analysis

All statistical analyses were performed using GraphPad Prism 9.5.1 (GraphPad Software, San Diego, USA) with data presented as the mean ± standard error of the mean (SEM). Statistical significance between two groups was evaluated using Student's *t*-test, while comparisons among multiple groups were conducted by one-way ANOVA followed by Bonferroni's* post hoc* test adjusted for multiple comparisons. A *P* value < 0.05 was considered statistically significant.

## Results

### Ele protects against diet-induced MASH in mice

**Figure 1:**
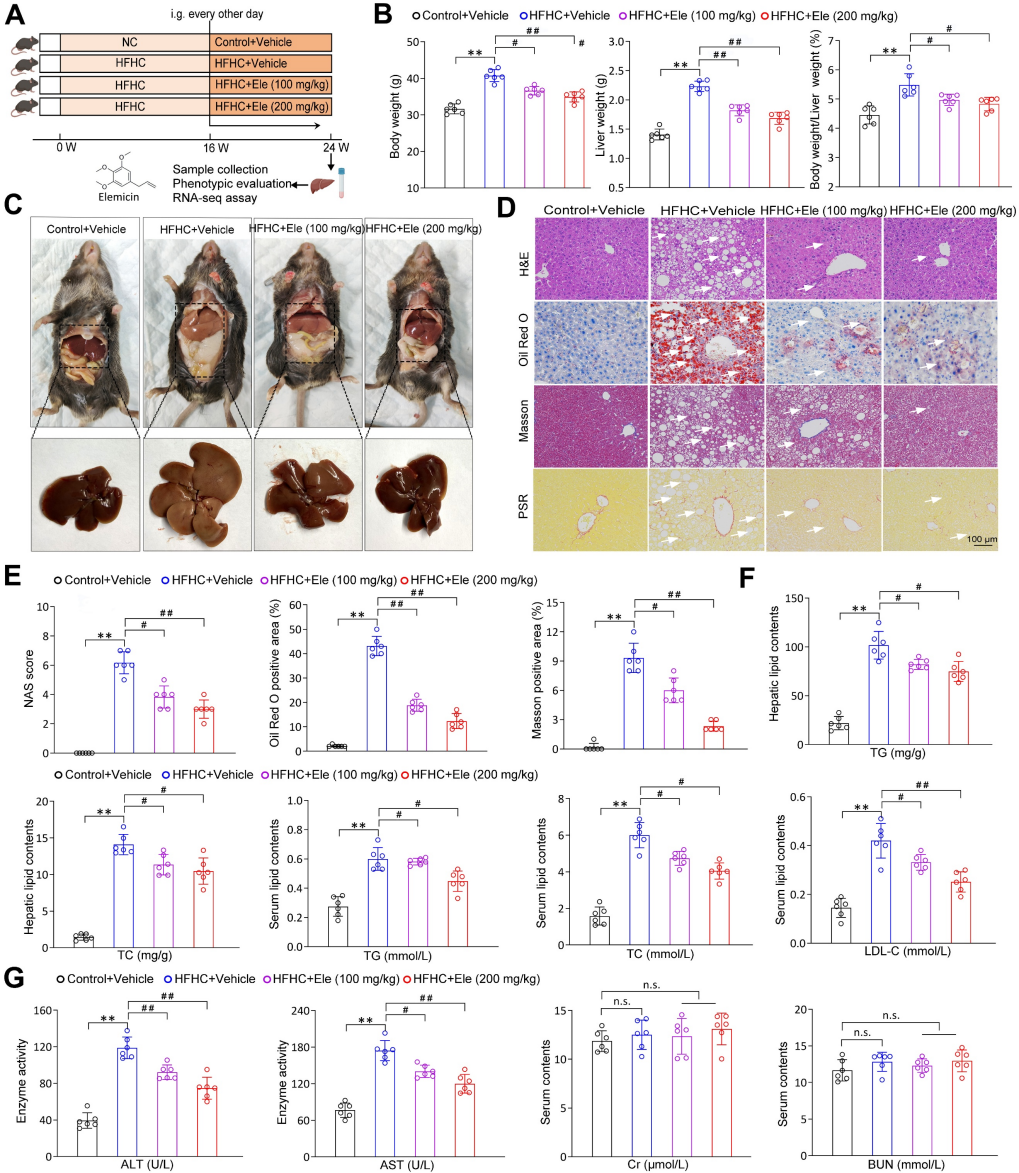
** Ele ameliorates HFHC-diet-fed hepatic steatosis, inflammation, and fibrosis in mice.** (A) Scheme illustrating Ele treatment strategy on a HFHC diet-induced MASH mouse model. Chemical structure of Ele. (B) Body weight, liver weight, and the ratio of liver weight to body weight of wild-type mice treated with Ele or vehicle at 24 weeks of HFHC or normal chow (NC) diet administration (mean ± SEM, *n* = 6). (C) Representative macroscopic and histological images of livers and liver sections (mean ± SEM, *n* = 6). (D) Representative H&E, oil red O, Masson, and PSR staining of liver sections from mice treated with vehicle or indicated doses of Ele (mean ± SEM, *n* = 6). (Scale bar, 100 μm.) NAS and quantitative results of oil red O- and Masson-positive areas are shown in (E) (mean ± SEM, *n* = 6). (F) Hepatic TG, TC, and serum TG, TC, and LDL-C concentrations in NC- or HFHC-diet-fed mice treated with either vehicle or Ele (mean ± SEM, *n* = 6). (G) Serum ALT, AST, Cr, and BUN levels of NC- or HFHC-diet-fed mice treated with vehicle or Ele (mean ± SEM, *n* = 6). (^*^P < 0.05, ^**^P < 0.01 *vs.* Control+Vehicle; ^#^*P* < 0.05, ^##^*P* < 0.01 *vs.* HFHC+Vehicle; n.s., not significant).

To directly assess the therapeutic potential of Ele in mitigating MASH progression, mice were fed a HFHC diet or a normal chow (NC) diet for 16 weeks to induce the MASH phenotype. Subsequently, intragastric gavage of Ele at 100 and 200 mg/kg/2 days was administered, concomitant with continued HFHC feeding for an additional 8 weeks (Figure 1A). Ele administration completely reversed the HFHC diet-induced increases in body weight, liver weight, and liver index, which exhibited no significant pathological changes in the NC diet group (Figure 1B-C). Moreover, the diagnosis of MASH was established using the NAS framework. Samples with a NAS ≥ 5, or with a NAS of 3-4 accompanied by fibrosis, were classified as MASH, whereas those with a NAS < 2 were defined as non-MASH. Histological assessment revealed exacerbated hepatic steatosis in HFHC-fed mice. This was collectively demonstrated by pronounced liver morphological alterations, hepatocellular injury featuring ballooning and microvesicular steatosis, significant lipid accumulation, and distinct lobular and pericellular fibrosis (Figure 1C-E). Ele administration presented remarkably ameliorated hepatic steatosis, inflammatory response, and fibrosis compared to vehicle treatment (Figure 1D-E). In addition, compared with the HFHC-vehicle group, Ele treatment markedly reduced collagen deposition and fibrotic area in 16-week HFHC mice (Figure S1A). These findings suggested that Ele prevented further progression of fibrosis while facilitating the regression of fibrotic tissue. Compared with the HFHC-vehicle group, mice treated with Ele displayed markedly reduced hepatic TG and TC levels, along with decreased serum TG, TC, and LDL-C concentrations (Figure 1F). In line with the above effect on HFHC-vehicle, diet-induced increases in serum ALT and AST levels were notably lower in Ele administration compared to the HFHC-vehicle, indicating accompanied by a mitigation in liver injury. Notably, in addition to its expected inhibition of hepatic biochemical indexes, Ele treatment did not exhibit a significant influence on the levels of renal biochemical indexes, including Cr and BUN; except for the liver, histological analyses revealed no evident additional pathological damage in the lung, kidney, heart, or spleen when compared to NC-vehicle (Figure 1G, Figure S1B). Collectively, pharmacological results demonstrate that Ele treatment is capable of alleviating major pathological features of MASH.

### Ele prevents MASH in mice by comprehensively suppressing lipid metabolism, inflammation, fibrosis, and apoptosis

**Figure 2:**
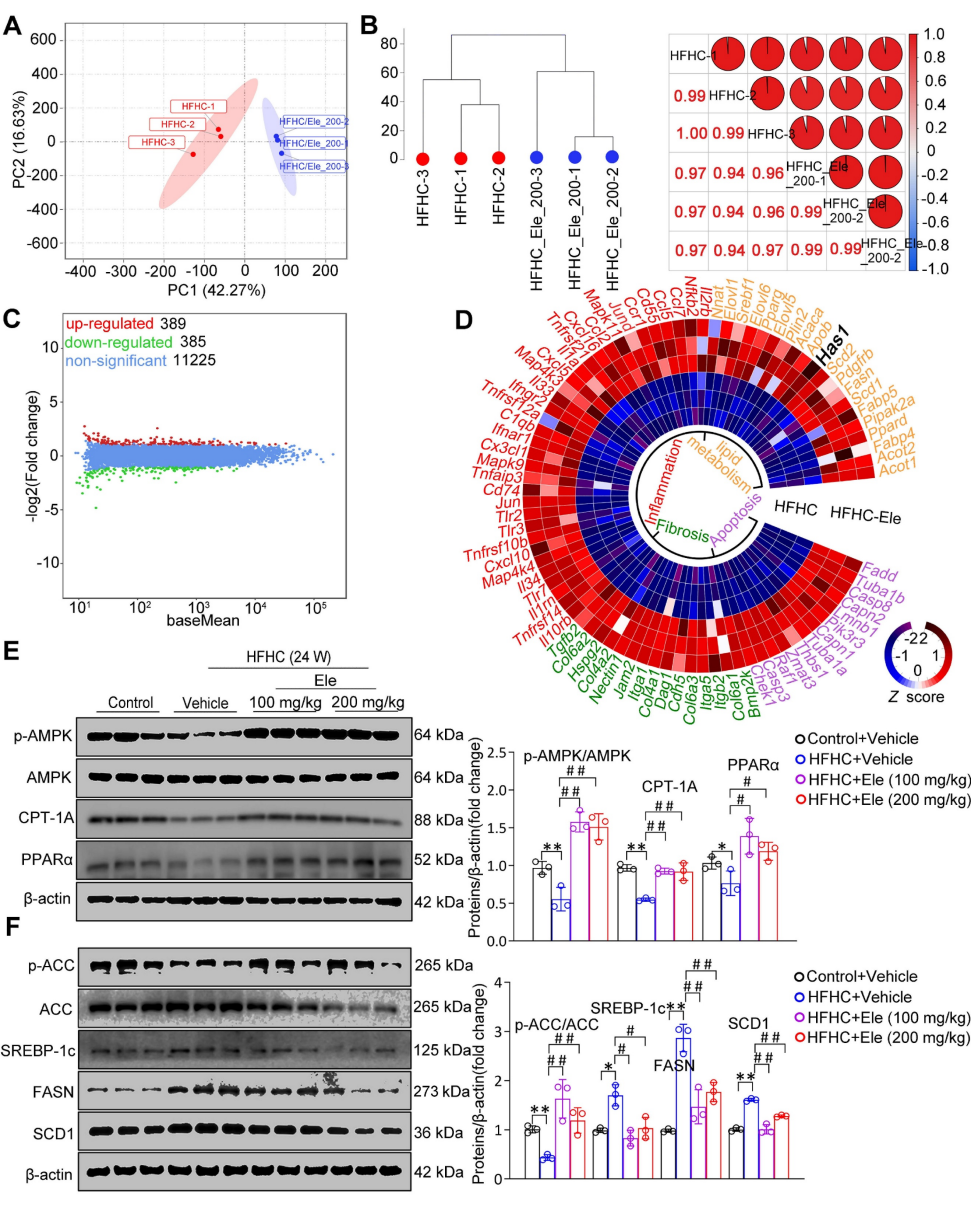
** Ele attenuates lipid metabolism in HFHC-diet-fed mice.** (A) PCA and (B) unsupervised hierarchical clustering analysis of the RNA-seq data from the mice treated with vehicle or Ele (*n* = 6). (C) The scatterplot depicting the fold change of all genes from RNA-seq analysis obtained from vehicle- or Ele-treated mice on NC or HFHC diet. Differentially expressed genes are highlighted in distinct colors (*n* = 6). (D) Radial heatmap illustrating the expression pattern of the top changed genes and enriched pathways in liver from vehicle- or Ele-treated groups on NC or HFHC diet. Western blot analysis and quantifications of total and phosphorylated AMPK, CPT-1A, and PPARα proteins (E), total and phosphorylated ACC, SREBP-1c, FASN, and SCD1 proteins (F) in livers from mice in the indicated groups (mean ± SEM, *n* = 3). β-actin served as a loading control (^*^P < 0.05, ^**^P < 0.01 *vs.* Control+Vehicle; ^#^*P* < 0.05, ^##^*P* < 0.01 *vs.* HFHC+Vehicle).

To systematically assess the specific impact of Ele at the molecular level, a comprehensive transcriptomic analysis was performed under HFHC diet-induced metabolic stress in liver tissues. Principal component analysis (PCA) and unsupervised hierarchical clustering distinctly separated the samples into two discernible clusters (Figure 2A-B). Among the differentially expressed genes identified by RNA sequencing between the livers of HFHC-vehicle and HFHC-Ele mice, Ele treatment upregulated 389 genes and downregulated 385 genes induced by HFHC (Figure 2C). As shown in Figure 2D, a heatmap based on the global gene expression profiles demonstrated a widespread downregulation of MASH-associated pathogenic pathways in HFHC-Ele livers relative to HFHC-vehicle controls. Gene set enrichment analysis (GSEA) systematically revealed that cellular signaling pathways related to lipid metabolism (such as the fatty acid biosynthetic process, steroid biosynthetic process, insulin signaling pathway, sphingolipid metabolism, and cholesterol biosynthetic process) were significantly enriched (Figure S1C). Therefore, protein expressions related to lipogenesis and lipolysis were investigated in HFHC-exposed mice. AMPK, as a crucial cellular energy sensor, inhibits lipogenesis and promotes lipolysis to help maintain the balance of lipid metabolism. After 24 weeks of HFHC treatment, results demonstrated that the phosphorylation of AMPK was suppressed in HFHC-vehicle mice, while Ele potently activated AMPK activity in HFHC-Ele mice (Figure 2E). ACC is a major substrate for AMPK and its phosphorylation status is typically assessed as an indicator of AMPK activation. Western blotting analysis demonstrated that Ele enhanced p-ACC/ACC, PPARα, and CPT-1A expression, while decreased SREBP-1c, FASN, and SCD1 levels compared with HFHC-vehicle group (Figure 2E-F).

**Figure 3:**
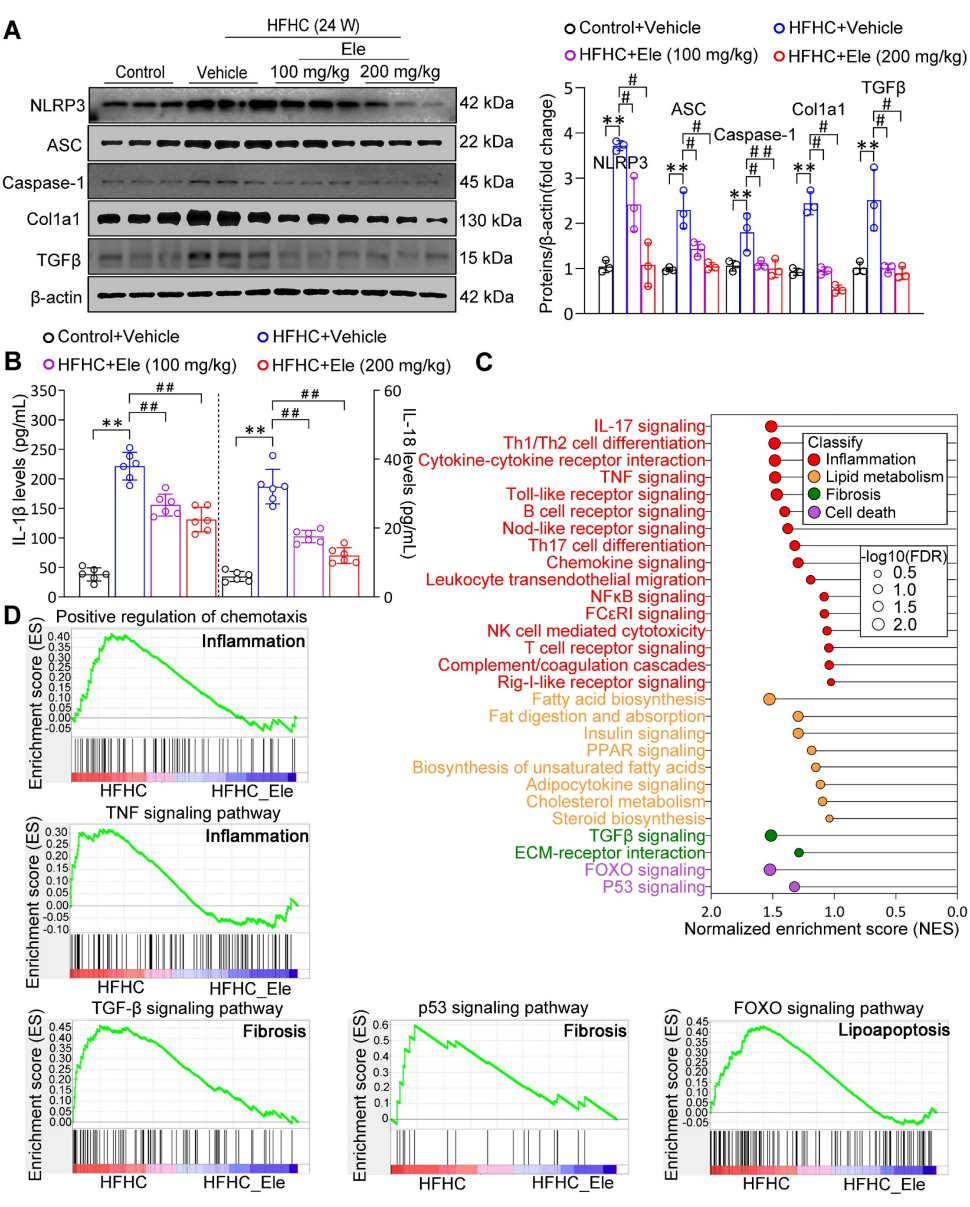
** Ele alleviates inflammation and hepatic fibrosis in HFHC-diet-fed mice.** (A) Western blot analysis and quantifications of NLRP3, ASC, Caspase-1, Col1a1, and TGFβ proteins in livers of mice treated with Ele or vehicle (mean ± SEM, *n* = 3). (B) Serum IL-1β and IL-18 concentrations in NC- or HFHC-diet-fed mice treated with either vehicle or Ele (mean ± SEM, *n* = 6). (C) Gene set enrichment analysis (GSEA) enrichment score, and (D) GSEA pathway enrichment results related to inflammation, lipid metabolism, fibrosis, and apoptosis from RNA-seq dataset on liver samples from mice in Ele treatment or vehicle control group (^*^P < 0.05, ^**^P < 0.01 *vs.* Control+Vehicle; ^#^*P* < 0.05, ^##^*P* < 0.01 *vs.* HFHC+Vehicle).

Given that the NLRP3 inflammasome serves as a pivotal mediator of inflammatory injury in MASH, exposure to the HFHC diet obviously upregulated the protein levels of NLRP3, ASC, and Caspase-1 (Figure 3A). Ele administration significantly reversed the changes, especially at 200 mg/kg (Figure 3A). Ele appears to act as a potential NLRP3 inhibitor. The activation of the NLRP3 inflammasome, a primary driver of IL-1β and IL-18 production in MASH, was effectively inhibited by Ele treatment, which correlated with reduced liver inflammation (Figure 3B). Importantly, GSEA data indicated a significant enrichment of genes associated with inflammation, lipid metabolism, fibrosis, and cell death among those influenced by Ele treatment (Figure 3C). Liver inflammation and fibrosis contribute to the exacerbation of MASH progression. GSEA unequivocally revealed that Ele administration suppressed key events related to inflammation (such as the positive regulation of chemotaxis and TNF signaling pathway), fibrosis (such as TGF-β signaling pathway and p53 signaling pathway), and cell death (such as FOXO signaling pathway) (Figure 3D). Taken together, genetic evidences strongly demonstrate that Ele exerts a potent protection against MASH progression.

### Ele attenuates PO-stimulated lipid accumulation, inflammation, fibrosis, and apoptosis in hepatocytes

**Figure 4:**
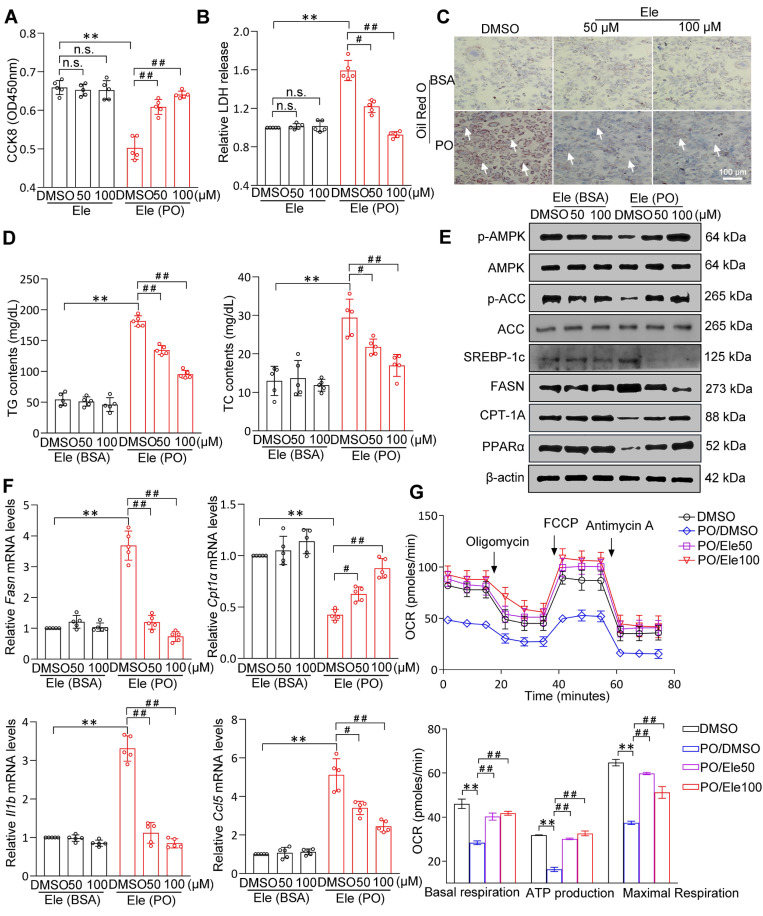
**The effect of Ele on fatty acid metabolism in hepatocytes.** Cell activity (A) and LDH release (B) detecting results in DMSO- or PO-induced mouse primary hepatocytes treated with vehicle and 50 or 100 μM of Ele as indicated for 24 h (mean ± SEM, *n* = 5). (C) Oil red O staining, (D) TG and TC content detection of primary hepatocytes treated with DMSO and Ele in response to PO stimulation (mean ± SEM, *n* = 5). (Scale bar, 100 μm.) (E) Representative western blot gels showing total and phosphorylated AMPK, total and phosphorylated ACC, SREBP-1c, FASN, CPT-1A, and PPARα proteins in primary hepatocytes in response to the corresponding stimulus. (F) The relative mRNA levels of *Fasn*, *Cpt1α*, *Il1b*, and *Ccl5* genes were determined by real-time PCR and normalized using *β-actin* as an internal control (mean ± SEM, *n* = 5). (G) Representative and parametric results of OCR in primary hepatocytes treated with DMSO or Ele (mean ± SEM, *n* = 3). Oligomycin, ATP synthase inhibitor; FCCP, mitochondrial uncoupler; Rotenone and antimycin A, complex I and III inhibitor, respectively (^*^P < 0.05, ^**^P < 0.01 *vs.* DMSO; ^#^*P* < 0.05, ^##^*P* < 0.01 *vs.* PO+DMSO).

Primary hepatocytes constitute the principal liver cell population and are central to the manifestation of metabolic disorders. Combined CCK8 and LDH cytotoxicity assays showed that Ele had no remarkable impact on cell viability (Figure 4A-B). PO resulted in significant lipid droplet formation and TG accumulation *in vitro* primary hepatocytes and as being indicated by ORO and Nile Red staining (Figure 4C, Figure S2A), which was supported by intracellular TG assay (Figure 4D). Consistently, after Ele treatment, Nile Red staining was blunted in PO-treated L02 hepatocytes (Figure S2B). In conclusion, both 50 and 100 μM of Ele dramatically ameliorated the steatosis phenotype induced by PO in cell. Western blotting analysis indicated a suppression in the levels of proteins associated with *de novo* fatty acid synthesis and an enhancement in those related to FAO and Ele markedly activated AMPK signaling in PO-treated hepatocytes (Figure 4E, Figure S2C-D). Quantitative PCR assays showed that Ele attenuated PO-mediated induction of fatty acid uptake and synthesis genes (*Fasn*, *Srebp*, *Hmgcr*, and* Acc*), as well as the mRNA expression levels of genes associated with inflammation (*Il1b*, *Il16*, *Ccl2*, *Ccl5*, *Tnfα*, and *Cxcl10*), were effectively diminished by Ele treatment (Figure 4F, Figure S3A). Additionally, the mRNA levels of lipid β-oxidation genes (*Pparα* and *Cpt1α*) were increased in Ele-treated primary hepatocytes (Figure 4F, Figure S3A). To further assess whether Ele affected FAO rate, cellular OCR assay showed that basal respiration, maximal respiration, and ATP production were significantly up-regulated by Ele treatment when compared to vehicle-PO treatment (Figure 4G), confirming that Ele administration robustly promoted FAO. However, glycolysis was suppressed in the presence of Ele treatment compared to the PO treatment, as indicated by ECAR in primary hepatocytes (Figure S3B). It is widely known that disruption of the tightly regulated glycolytic and lipogenic networks is considered a possible factor underlying MASH progression. Based on metabolic flux analysis, OCR and ECAR clearly indicate that Ele supplementation leads to a shift in the lipid metabolic characteristics in primary hepatocytes induced by PO, encompassing the inhibition of glycolysis and a concurrent augmentation in FAO.

### Hepatocyte-specific Has1 deficiency ameliorates major features of MASH in mouse primary hepatocytes

**Figure 5:**
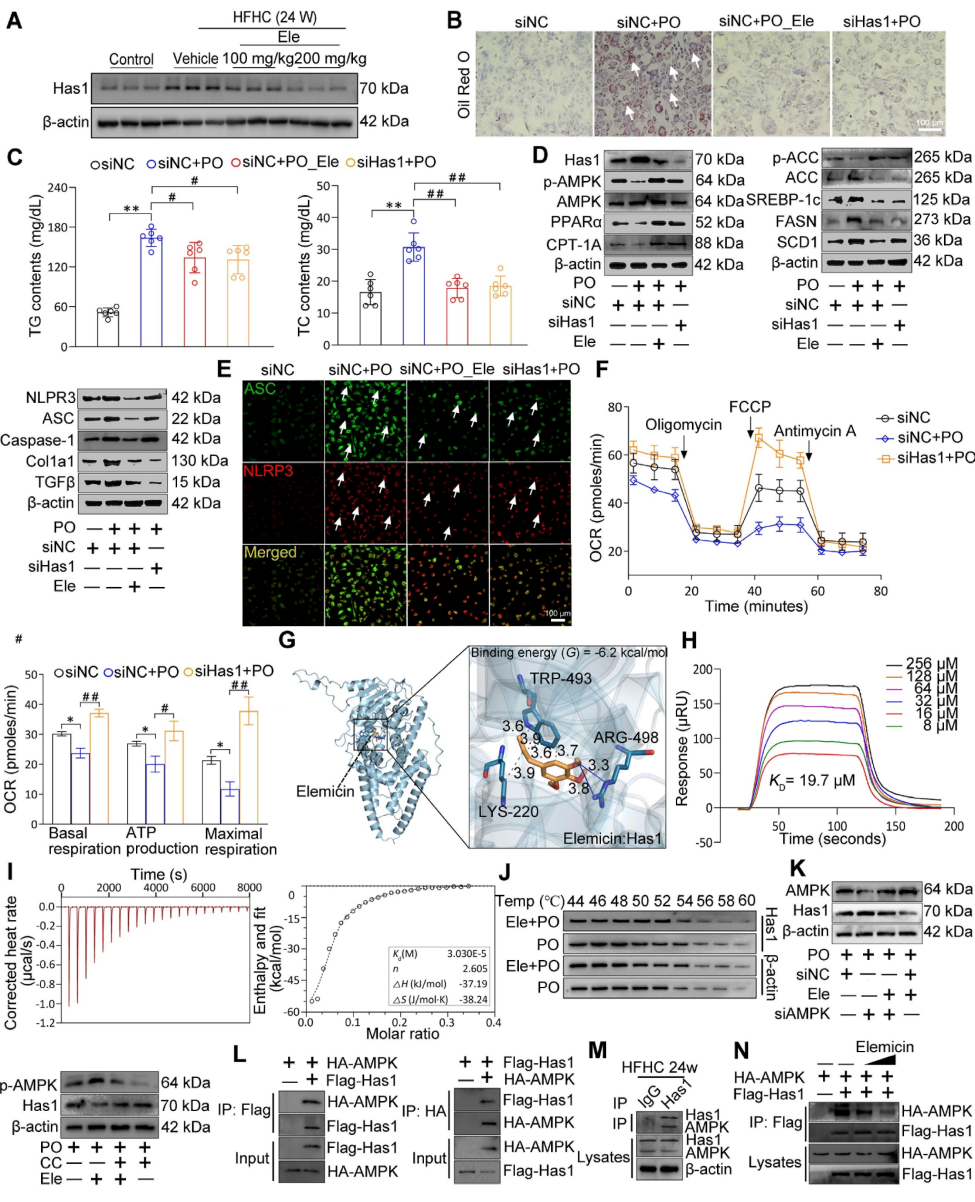
** Specific Has1 knockdown protects from PO-induced MAFL and MASH in hepatocytes.** (A) Representative western blot gels of Has1 protein in livers from mice treated with vehicle or indicated doses of Ele (mean ± SEM, *n* = 3). (B) Oil red O staining, (C) TG and TC content detection of primary hepatocytes in response to PO stimulation treated with/without Ele or siRNA against Has1 (mean ± SEM, *n* = 5). (Scale bar, 100 μm.) (D) Representative western blot gels showing Has1, total and phosphorylated AMPK, PPARα, CPT-1A, total and phosphorylated ACC, SREBP-1c, FASN, SCD1, NLRP3, ASC, Caspase-1, Col1a1, and TGFβ proteins in primary hepatocytes in response to the corresponding stimulus treated as described in* C*. (E) Representative immunofluorescence staining of ASC (green) and NLRP3 (red) in primary hepatocytes with indicated treatments. (Scale bar, 100 μm.) (F) Mitochondrial bioenergetic analysis of primary hepatocytes in response to PO stimulation treated with siRNA against Has1 (mean ± SEM, *n* = 3). Binding affinity and kinetic parameters for the interactions between Ele and Has1 protein. (G) Docking images showing Has1 and binding position of Ele at Has1 protein, and (H) kinetic analysis using SPR for Ele binding to Has1. (I) ITC assay showing direct interaction of Has1 protein and Ele (mean ± SEM, *n* = 3). (J) CETSA analysis of the binding specificity of Ele with Has1 protein in PO-induced hepatocytes under the indicated conditions. (K) Western blotting in primary hepatocytes transduced with an AMPK protein expression vector (siAMPK). Cells transduced with empty vector (siNC) served as control. The expression levels of p-AMPK and Has1 were determined after co-treatment with CC. (L) Results by Co-IP assays in primary hepatocytes transfected with Flag-tagged Has1 and HA-tagged AMPK. Anti-Flag and anti-HA antibodies were used as immunoblotting probes. (M) Immunoprecipitation and western blotting analysis indicating the binding of Has1 and AMPK in liver WT mice after a 24-week HFHC, and IgG was served as a control. (N) Representative gels showing the influence of Ele on interaction between Flag-labeled Has1 and HA-labeled AMPK in 293T cells examined by immunoprecipitation and western blot assay (^*^P < 0.05, ^**^P < 0.01; ^#^*P* < 0.05, ^##^*P* < 0.01).

Given the preceding radial heatmap, which illustrated that Has1, a core enzyme in the lipid metabolic pathway, was among the most significantly downregulated genes in the HFHC-Ele group in terms of fold change compared to the HFHC group, these findings suggested its potential as a conserved gene with greater contribution (Figure 2D, Figure S4A). Protein and mRNA abundances of Has1 were dramatically elevated in liver issues from mice with MASH, and Ele (100 and 200 mg/kg) exhibited an observable inhibitory effect (Figure 5A, Figure S4B-C). To specify the contribution of hepatic Has1 in the pathogenesis of MASH, Has1 gene was knocked down in hepatocytes through transduction with siRNA and subjected the cells to PO treatment. Has1 deficiency prominently inhibited lipid accumulation in response to PO challenge compared with the control group, as evidenced by ORO staining and intracellular TG assay (Figure 5B-C, Figure S4D). Importantly, Has1 deficiency potently induces PO-linked protein expression changes in hepatocytes. Our previous studies showed that the pharmacological reactivation of down-regulated AMPK in high diet-fed mice could effectively normalize hepatic lipid contents through inhibition of DNL and promotion of FAO 17,18. Therefore, the association between Has1 and AMPK was initially observed. After PO incubation, western blotting results confirmed that silencing Has1 markedly enhanced AMPK phosphorylation in primary hepatocytes, accompanied by regained lipolysis, weakened lipogenesis, and reduced proinflammatory response (Figure 5D, Figure S4E). Given that hepatic stellate cells (HSCs) are the primary producers of extracellular matrix in liver fibrosis, we established a non-contact co-culture system of L02 hepatocytes and LX-2 cells to study their interactions. PO-stimulated L02 hepatocytes significantly induced the activation of LX-2 cells in co-culture, evidenced by a strong upregulation of Col1a1 and α-SMA expression (Figure S4F). However, when Ele was applied on the hepatocyte side or Has1 was knocked down in hepatocytes, the activation of LX-2 cells was significantly suppressed (Figure S4G). Notably, adding Ele directly to the LX-2 cell chamber did not yield a significant inhibitory effect. These results indicate that Ele targeted Has1 in hepatocytes to modulate the secretion of hepatocyte-derived pro-fibrotic signals, thereby indirectly inhibiting HSC activation. ASC specks, acting as inflammasome adapters, have the capability to amplify the activation of NLRP3 inflammasomes and exacerbate the progression of MASH. Up-regulated ASC and NLRP3 intensities were observed throughout primary hepatocytes as the PO induction progressed. However, siHas1 treatment markedly attenuated the PO-induced formation of ASC and NLRP3 in primary mouse hepatocytes compared to the siNC controls (Figure 5E, Figure S4H). Additionally, Has1 deficiency significantly augmented the intrinsic capacity of mitochondrial respiration in Has1 silenced hepatocytes, as evidenced by the metabolic fitness OCR (Figure 5F).

To verify the interaction mode between Ele and Has1, we performed protein-ligand docking and derived possible binding sites. The predicted binding mode revealed a good shape match between Ele and the binding pocket residues Arg498 of Has1 were involved in forming hydrogen bonds with Ele, whereas Trp493 and Lys220 were involved in hydrophobic contacts with Has1 (Figure 5G). In addition, the free binding energy of Ele and Has1 protein was -6.2 kcal/mol, indicating that Ele can spontaneously and stably bind with Has1 protein (Figure 5G). These computational results indicate that Ele has a strong binding affinity for Has1, supporting the potential for a direct molecular interaction. These findings provide preliminary evidence for a possible interaction between Ele and Has1, which warrants further experimental validation to clarify its functional role. Furthermore, SPR analysis further verified that Ele bound directly to Has1 in a dose-dependent manner, displaying strong affinity with a dissociation constant (*K_D_*) of 19.7 μM according to steady-state analysis (Figure 5H). Consistent with the SPR-derived *K_D_* value, Ele at 20 μM significantly reduced lipid accumulation (Figure S4I), supporting its specificity within a pharmacologically relevant concentration range. Using an ITC assay, we demonstrated that Ele directly bound to Has1 (Figure 5I). CETSA assays found that Has1 protein alone denatured approximately 54 ~ 56 °C within the cells, and Has1 protein was stabilized by 2 ~ 4 °C upon binding with Ele. Collectively, the elevated thermal stability of Has1, evidenced by its increased denaturation temperature, identifies Ele as a direct binding partner that stabilizes the protein (Figure 5J). As our previously demonstrated, AMPK functioned as a highly conserved central regulator of cellular metabolism, facilitating lipid catabolism upon activation in MASH 17. As shown above, Ele also increased the phosphorylation of AMPK *in vivo* and *in vitro*. To further verify that the association between AMPK and Has1, hepatocytes were transfected with AMPK-specific siRNA or siNC, and then co-treated with PO in the presence or absence of Ele.

Notably, Ele treatment attenuated the siAMPK-induced reduction of p-AMPK, indicating that Ele is capable of restoring AMPK activation under AMPK-deficient conditions (Figure 5K, Figure S4J). Consistently, pharmacological inhibition of AMPK using Compound C (CC) significantly counteracted the Ele-mediated downregulation of Has1 expression. (Figure 5K, Figure S4J). Together, these findings demonstrate that AMPK activation is mechanistically required for Ele-dependent regulation of Has1. Accordingly, Co-IP analysis revealed a direct molecular interaction between Has1 and AMPK (Figure 5L), which was further validated in PO-challenged primary hepatocytes (Figure 5M). However, Ele treatment markedly disrupted with the AMPK-Has1 interaction (Figure 5N, Figure S4K). The above results suggest that targeting the formation of AMPK/Has1 complex can selectively reduce AMPK activity. These data validated that Ele interrupted the AMPK-Has1 interaction to attenuate the principal pathological features of MASH and established that Has1 was a key regulator of MASH progression.

### Liver Has1 inhibition ameliorates HFHC-induced MASH pathologies

**Figure 6:**
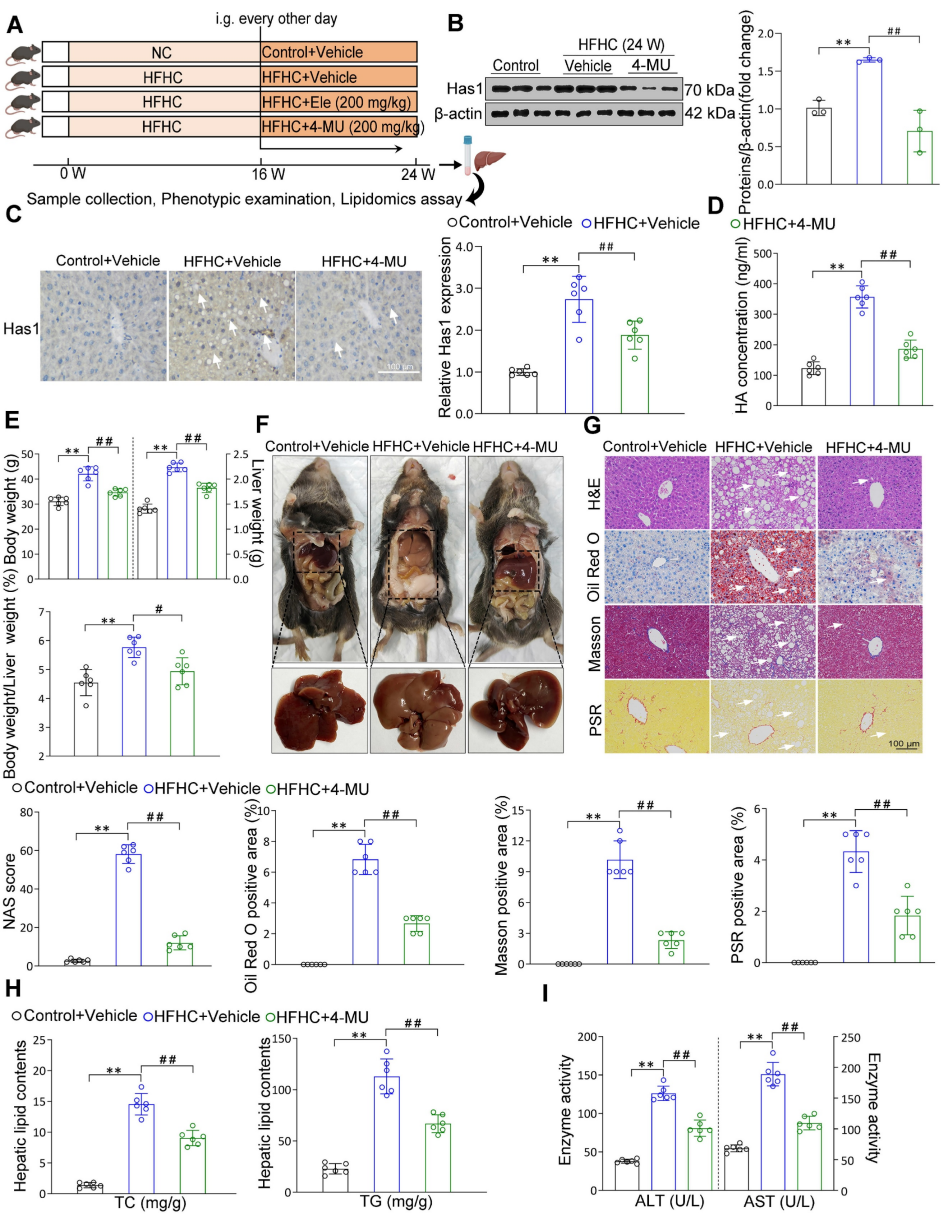
**4-MU effectively inhibits MASH progression in diet-induced mice.** (A) Flow chart showing strategy for evaluating 4-MU function on mice with spontaneously developed MASH that was exacerbated by HFHC treatment. Representative western blot gels and quantification (B), and IHC staining and quantification (C) of serial liver sections from HFHC-fed mice in indicated groups showing the Has1 protein (mean ± SEM, *n* = 3 in western blot and *n* = 6 in IHC). (Scale bar, 100 μm.) (D) Serum hyaluronan content detection in response to HFHC stimulation treated with/without 4-MU (mean ± SEM, *n* = 5). (E) Body weight, liver weight, and the ratio of liver weight to body weight of wild-type mice treated with 4-MU or vehicle at 24 weeks of HFHC or NC diet administration (mean ± SEM, *n* = 6). (F) Representative macroscopic and histological images of livers and liver sections (mean ± SEM, *n* = 6). (G) Representative H&E, oil red O, Masson, and PSR staining of liver sections from mice treated with vehicle or indicated doses of Ele (mean ± SEM, *n* = 6). (Scale bar, 100 μm.) NAS and quantitative results of oil red O-, Masson- and PSR-positive areas are also shown in (G) (mean ± SEM, *n* = 6). Hepatic TG and TC (H), and serum ALT and AST (I) in NC- or HFHC-diet-fed mice treated with either vehicle or 4-MU (mean ± SEM, *n* = 6). (^*^P < 0.05, ^**^P < 0.01 *vs.* Control+Vehicle; ^#^*P* < 0.05, ^##^*P* < 0.01 *vs.* HFHC+Vehicle).

Based on the associations between liver Has1 levels and MASH, the specific role of Has1 was evaluated using its inhibitor of 4-methylumbelliferone 19 (4-MU, 200 mg/kg) supplemented to mice on an HFHC diet (Figure 6A). Under a 24-week HFHC feeding, Has1 abnormal level was inhibited by the 4-MU (Figure 6B). Moreover, 4-MU treatment also remarkably reduced hepatic Has1 activity (Figure 6C). In addition, we also examined the roles of Has2 and Has3 isoforms. Isoform-selective knockdown in hepatocytes indicated that silencing Has2 or Has3 did not produce significant changes in α-SMA or CPT-1A proteins (Figure S5A). Consistently, Ele treatment preferentially reduced Has1 expression (Figure S5B), suggesting that Has1 is the predominant isoform involved under our experimental conditions. Hyaluronan (HA), synthesized by Has1, has been implicated in metabolism-associated diseases, and alterations in its levels have been shown to promote the secretion of proinflammatory cytokines during MASH 19. Its abnormal synthesis level was also inhibited by the 4-MU supplementation (Figure 6D). The HFHC-4MU group demonstrated a decrease in key hepatic weight parameters relative to the HFHC-vehicle group, and the intensified liver appearance changes were observed to attenuate (Figure 6E-F). Additionally, histological examination of liver sections showed a spontaneous hepatic phenotype related to MASH that hepatic lipid deposition, ballooning degeneration, inflammatory cell infiltration, and liver fibrosis were all evidently weakened by 4-MU administration relative to the HFHC-vehicle group (Figure 6G). Continuously, NAS score was alleviated as well. The comparable hepatic triglyceride lipid (TC and TG) levels, the serum levels of ALT and AST between HFHC-vehicle and HFHC-4-MU mice indicated that 4-MU treatment alleviated hepatic lipid deposition and liver injury (Figure 6H-I). Given the important roles of Has1 in mice subjected to HFHC feeding had been preliminarily defined in the study, Has1 mRNA expression was notably elevated in MASH patients than in controls in the GEO database GSE48452 (n = 45 *vs.* 43). Consistently, validation in an independent large cohort (GSE89632, n = 63) confirmed a marked upregulation of Has1 in MASH livers in agreement with our findings (Figure S5C). Meanwhile, measurement of serum HA levels revealed a significantly higher concentration in the 7 MASH patients compared to the 6 healthy controls (Figure S5D). Moreover, the IHC results showed a remarkable upregulation of Has1 in the liver of MASH patients (Figure S5E). Consistently with 4-MU, a remarkable reduction in total HA content was observed following treatment with Ele (Figure S5F). Hepatic inflammatory and fibrosis were markedly ameliorated by Has1 deficiency (Figure S5G, top), and AMPK activation had beneficial effects on fatty acid synthesis and oxidation (Figure S5G, bottom). AMPK expression was observed to decrease in MASH patients versus healthy controls, as evidenced by the GEO database (Figure S5H), suggesting that AMPK could act as a potential prognostic indicator and may also be strongly correlated with Has1 in the context of MASH. These data validated that pharmacological inhibition of Has1 expression attenuated the principal pathological features of MASH, and the magnitudes of the anti-MASH effects elicited by genetic deficiency of Has1 *in vitro* largely mimicked the effects of the pharmacological inhibitor of 4-MU* in vivo*.

### Ele alters hepatic lipidomics during the treatment of MASH

**Figure 7:**
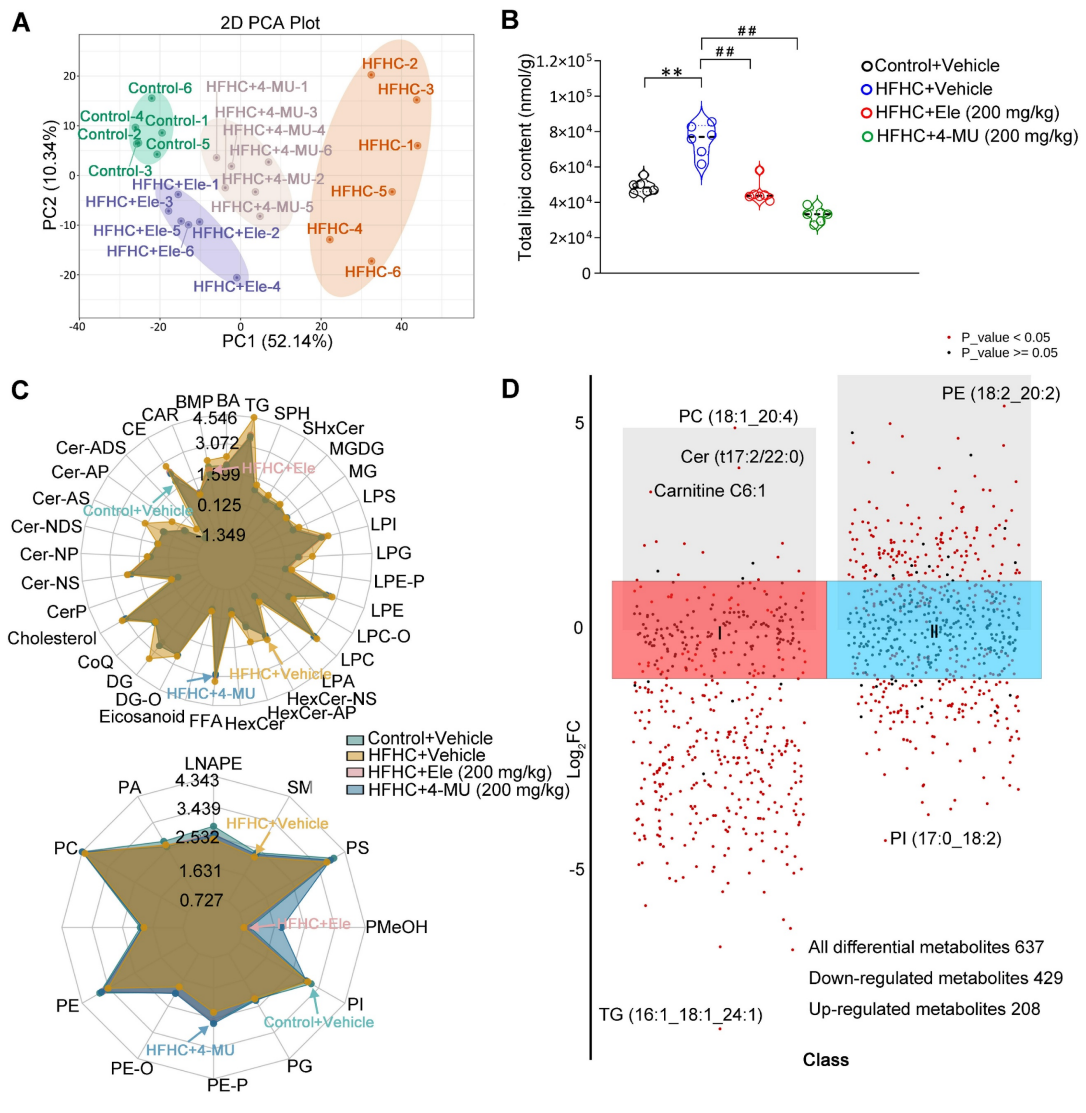
**Lipidomics analysis of liver tissues in MASH.** PCA score plot of the hepatic lipid profiles (A) and total lipid content (B) of livers treated with 4-MU or vehicle at 24 weeks of HFHC or NC diet administration (mean ± SEM, *n* = 6). (C) Radar chart showing the difference in the total content of different lipid subclasses. (D) DALs analysis showing up- and down-regulated lipids across all two clusters. An adjusted *p* value < 0.05 is indicated in red, while an adjusted *p* value ≥ 0.05 is indicated in black. (^*^P < 0.05, ^**^P < 0.01 *vs.* Control+Vehicle; ^#^*P* < 0.05, ^##^*P* < 0.01 *vs.* HFHC+Vehicle).

To further verify the effect of Ele and 4-MU on systemic changes in hepatic lipid metabolism in the HFHC-induced mouse MASH model, a quantitative lipidomic analysis of mouse livers was employed to assess the transformed intrahepatic lipid profile in HFHC, Ele, and 4-MU treatment groups versus the control. The unsupervised PCA scores showed a distinctive classification trend between control and HFHC groups, with the Ele and 4-MU group exhibiting closer proximity to the control group (Figure 7A). The results highlighted a significant difference in the lipid metabolite levels between control and HFHC groups. Following the administration of Ele and 4-MU, these levels showed a tendency to normalize, suggesting that Ele and 4-MU had a beneficial effect in reducing the abnormal lipid metabolites in HFHC-fed mice. Consistently, a significant reduction in total lipid content during the occurrence and treatment of MASH was observed following treatment with Ele and 4-MU (Figure 7B). To characterize the regulatory preference of Ele on hepatic lipid metabolism, we systematically visualized the abundance of differentially altered lipid metabolites (DALs), which were categorized based on the LIPID MAPS classification (lipidmaps.org). In general, lipids can be broadly classified into eight major groups: fatty acyls (FA), glycerolipids (GL), sterol lipids (ST), sphingolipids (SP), glycosphingolipids (SoG1), glycerophospholipids (GP), prenolipids (PR), and polyketides (PK) 20. As the main lipid classes in mammals, unsurprisingly, the overall content of FA, GL, SP, and ST induced by HFHC-diet significantly decreased following supplementation with Ele (Figure 7C, top), and this observation aligned with the characteristic feature of MASH, marked by excessive intrahepatic fat deposition 21. The alterations in hepatic lipidomic profiles, in turn, contributed to mitigating hepatocyte dysfunction, inflammation, and fibrosis. Conversely, the content of GP markedly increased (Figure 7C, bottom), with the primary constituents including PE, PC, PS, PA, and PI, while there was no significant change observed in PR, SoG1 and PK. The scatter plot of DALs also sharply illustrated lipid variations in the content of different lipid subclasses and shifted the profile of lipids in the HFHC diet-fed liver (Figure S6A-B). A total of 637 DALs were detected in the lipidomic analysis and significant differences were noted in 474 DALs between the HFHC group and Ele group, out of which 429 were down-regulated and 20 up-regulated (Figure 7D, Figure S6C). Consistent with liver pathology, the bar chart of DALs demonstrated that the most significant and substantial decreases in lipids associated with MASH were presented in TGs (Figure S6D), which positively improved the TGs profiles. These results indicated that both Ele and 4-MU effectively alleviated HFHC-induced hepatic steatosis, and Ele exhibited different preferences in modulating lipid profiles. To investigate whether the lipidomic effects of Ele, a reported SCD1 inhibitor 22, are mediated by SCD1 inhibition, we conducted parallel experiments with the selective SCD1 inhibitor MK-8245 (Figure S6E), which showed no effect on Has1 expression or AMPK activation. In contrast, Ele significantly downregulated Has1 and enhanced p-AMPK levels. Additionally, SCD1 overexpression did not reverse the effects of Ele on Has1 suppression or AMPK activation (Figure S6F). These findings indicate that the lipidomic changes induced by Ele are primarily mediated through a Has1/AMPK-dependent pathway, rather than through SCD1 inhibition.

### Effect PE on the occurrence and treatment of MASH

**Figure 8:**
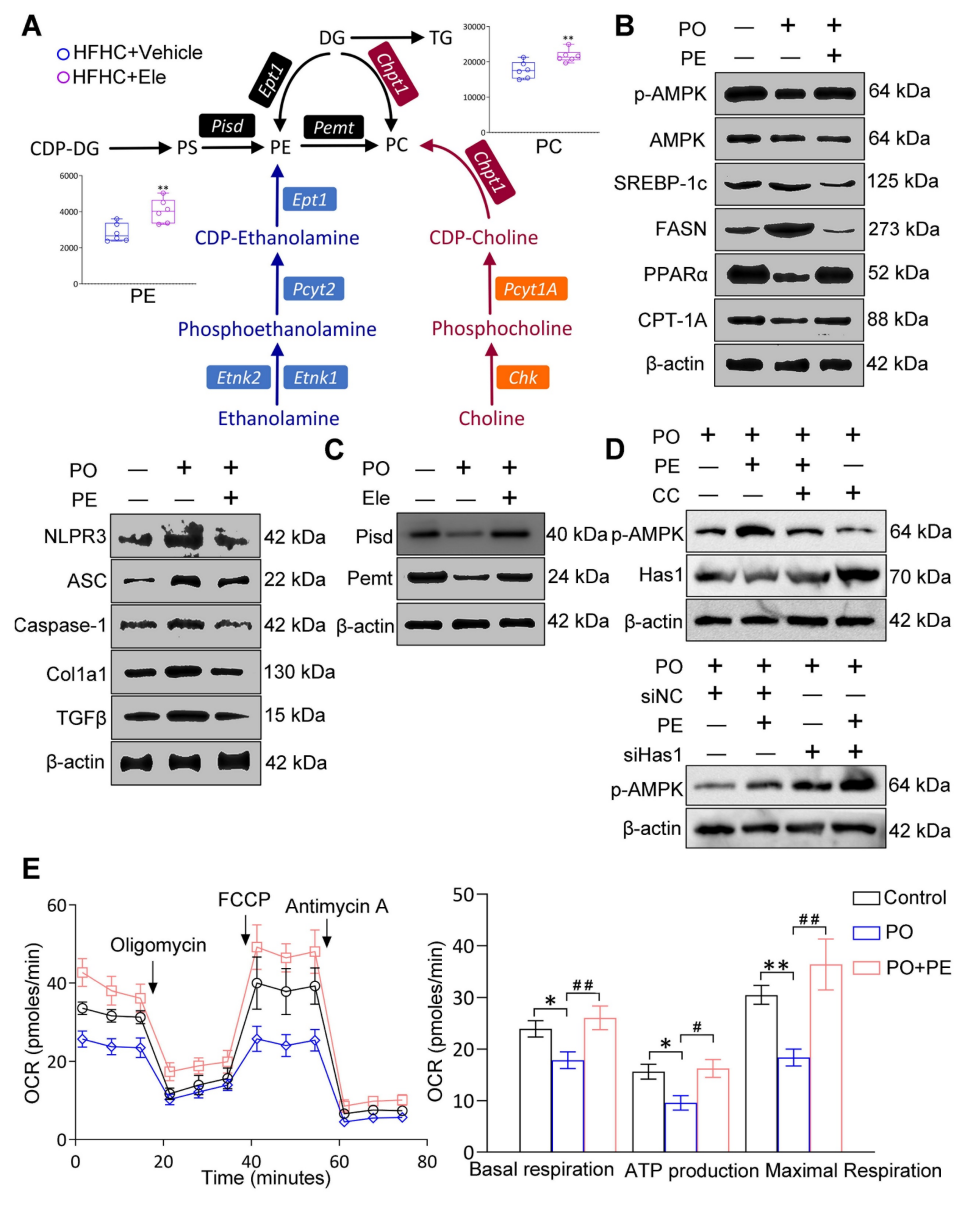
** The potential factors that MASH and Ele regulate PE on HFHC diet-induced metabolic dysfunction-associated steatohepatitis in mice.** (A) The main pathways of intrahepatic PE and PC biosynthesis and metabolism following Ele treatment in HFHC diet-fed mice (mean ± SEM, *n* = 6). (B and C) Western blot analysis of total and phosphorylated AMPK, SREBP-1c, FASN, PPARα, CPT-1A, NLRP3, ASC, Caspase-1, Col1a1, TGFβ, Pisd, and Pemt proteins in primary hepatocytes in response to PO stimulation treated with PE. (D) Western blot analysis of p-AMPK and Has1 expression following treatment with PE in combination with CC or siHas1. (E) Representative and parametric results of OCR in primary hepatocytes in response to the corresponding stimulus treated with PE (mean ± SEM, *n* = 3). (^*^P < 0.05, ^**^P < 0.01 *vs.* DMSO; ^#^*P* < 0.05, ^##^*P* < 0.01 *vs.* PO+DMSO).

Generally, lipidomics can elucidate the fundamental role of dynamic lipid metabolism in disease pathogenesis, thereby contributing to a comprehensive understanding of disease pathogenesis. Correlation analysis revealed that metabolites such as FFA, TG, and PG displayed antagonistic relationships, whereas multiple PE species exhibited strong positive correlations, indicative of a synergistic effect (Figure S7A-B). These correlation patterns aligned with our biochemical and histological findings, supporting the conclusion that Ele intervention reprogrammed hepatic lipid metabolic networks and this also aided in quantifying the metabolic proximities between DALs. Given the striking discovery in this study was that Ele treatment markedly elevated intrahepatic PE and PC levels in mice fed an HFHC diet, and we also summarized the principal alterations in biosynthetic and metabolic pathways following Ele supplementation (Figure 8A). Considering the variations in hepatic lipidomics, PE demonstrates high abundance, is commercially available, and correlates with MASH progression and Ele intervention. We hence aimed to determine whether PE functions as the targeted lipid on MASH using *in vitro* models. In this process, primary hepatocytes induced by PO were treated with 20 μM of PE and then the protein expressions associated with MASH were firstly measured. Meanwhile, the protein levels related to lipid metabolism (including p-AMPK/AMPK, CPT-1A, PPARα, FASN, and SREBP-1c), inflammation and fibrosis (including NLRP3, ASC, Caspase-1, Col1a1, and TGFβ) were notably reversed these alterations in PO-administrated group (Figure 8B, Figure S7C). It is well-known that PC and PE, the two major eukaryotic GP, share a biosynthetic dependence on the formation of CDP-choline and CDP-ethanolamine for their production, which are involved in the respective synthesis processes of PE and PC. To investigate how MASH and Ele influence PE levels, we examined the expression of key enzymes responsible for PE synthesis and metabolism, including Pisd and Pemt, which were significantly increased in Ele-treated hepatocytes (Figure 8C, Figure S7D).

Furthermore, PE treatment significantly promoted AMPK phosphorylation and suppressed Has1 expression in hepatocytes, effects which were attenuated by co-treatment with CC (Figure 8D, Figure S7E). In addition, the combination of Has1 silencing and PE supplementation synergistically enhanced AMPK phosphorylation (Figure 8D, Figure S7E). Collectively, these findings demonstrate that the beneficial effects of PE are mediated through the AMPK-Has1 signaling axis. MASH drives mitochondrial lipid remodeling, subsequent to a reprogramming of mitochondrial energy metabolic phenotypes. So, to evaluate the effects of PE on mitochondrial function, OCR measurements were performed, showing that basal respiration, ATP production, and maximal respiration were significantly up-regulated, which implied that PE treatment might facilitate FAO (Figure 8E). Obviously, PE administration had evidently ameliorated lipid metabolic disorder, inflammation, and fibrosis in an *in vitro* model of MASH, and could restore homeostasis of mitochondrial energy metabolic phenotype. PE might be a promising reliable biomarker for MASH, and its impact on MASH will be further validated in future study using HFHC diet-fed mice.

## Discussion

Current epidemiology points to MASH as a primary driver of liver dysfunction globally. An inadequate comprehension of the complicated pathogenic mechanism underlying MASH has significantly impeded the advancement of drug development for MASH. There currently exists a dearth of acknowledged and approved pharmacological interventions for the treatment of MASH except the cornerstone of long-term management, lifestyle modifications. In the present study, we have demonstrated that obese mice subjected to an HFHC diet exhibit accelerated progression towards severe obesity, NAFLD, and MASH. Furthermore, by applying transcriptomic and lipidomic analysis, we systemically elucidate that phytochemical Ele possesses the capability to ameliorate hepatic steatosis, inflammation, and liver fibrosis in HFHC-fed mice *via* the interruption of the formation of AMPK-Has1 complex.

Recent evidence highlights the pivotal role of phytochemicals in preventing and treating MASH, providing insights into etiology, pathogenesis, diagnosis, and therapeutic interventions, which prompts researchers to explore safe and effective natural products, especially given the extremely long duration of modern drug discovery processes 22,23. Ele, as a dietary supplement, possesses pharmacological effects such as antioxidant, anti-inflammatory, and improvement of metabolic disorders 15,16. In a long-term HFHC-induced MASH mouse model, we found that Ele administration prominently alleviated the exacerbated hepatic steatosis, inflammation, and fibrosis, as indicated by reduced hepatocyte ballooning area, blocked collagen fiber accumulation, lowered intrahepatic and serum TG/TC levels, and ameliorated liver injury, as evidenced by reduced serum AST/ALT levels. Although further validation in advanced fibrosis models remains a critical future direction, our current data indicated that facilitated the reversal of existing fibrotic deposits. Furthermore, no noticeable side effects were observed. Previous toxicology studies indicate that Ele, under certain exposure conditions, produces liver effects 24,25. Several factors likely account for the apparently contrasting findings between those reports and the present study. Pathophysiological context may alter response: mice with diet-induced MASH exhibit marked changes in hepatic metabolism and gut microbiota that could modulate both therapeutic and adverse responses. Despite toxicity at 200 mg/kg in healthy animals, the improvement of MASH phenotypes at 100 and 200 mg/kg suggests a potential disease-specific therapeutic window. Although preliminary organ histology and *in vitro* cytotoxicity studies indicate that Ele is well tolerated, we recognize that further comprehensive investigations, including preclinical pharmacokinetic and toxicological assessments, are essential to fully evaluate its safety and clinical relevance in future studies. To uncover the potential mechanisms by which Ele mitigates MASH, transcriptome analysis followed by functional validation revealed that Ele supplementation substantially reversed HFHC diet-induced detrimental gene expression patterns. Enrichment analysis further indicated that the genes modulated by Ele treatment were predominantly associated with lipid metabolism, inflammation responses, and fibrogenesis. More importantly, Has1 was maybe identified as a critical suppressor of MASH and was potentially both necessary and sufficient for anti-MASH.

AMPK, a central nutrient and energy sensor crucial for maintaining energy homeostasis, its activation has long been suggested to hold therapeutic benefits in the context of metabolic diseases 26-28. AMPK is markedly phosphorylated to regulate a spectrum of biochemical responses, including the suppression of anabolic pathways such as fatty acid and cholesterol synthesis, and the promotion of catabolic pathways like FAO 6. Accumulating evidence has convincingly shown that AMPK loss aggravates MASH pathology in diet-induced models, and AMPK activation is required for therapy in MASH 29-31. Consistent with this association, our current data indicated that inhibition of hepatic AMPK accelerated lipid accumulation in the livers of HFHC-fed mice, while pharmacological reactivation of downregulated AMPK by Ele effectively normalized hepatic lipid content in fatty liver. Hepatocytes are primarily responsible for the major physiological and metabolic functions of the liver, yet a central driver in the onset of MASH the metabolic imbalance between DNL and FAO in hepatocytes 32-34. Mice on HFHC-inducing diet appeared dysregulation of metabolic enzymes associated with downstream of AMPK, including enzymes related to DNL, such as p-ACC, SREBP-1c, FASN, and SCD1, (*e.g.*, p-ACC, SREBP-1c, FASN, and SCD1), as well as FAO enzymes, including PPARα and CPT-1A. These alterations were significantly ameliorated by the intragastric administration of Ele. MASH is pathologically defined by hepatic inflammation, which involves two critical events: damage to hepatocytes and the consequent release of proinflammatory cytokines 35. In this study, exposure to the HFHC diet notably upregulated the expression of NLRP3 inflammasome components, including NLRP3, ASC, and Caspase-1, which serves as a crucial mediator of hepatic inflammatory responses in MASH. IL-1β and IL-18, as pleiotropic cytokines involved in inflammatory responses, activated by NLRP3 inflammasome, are also both implicated in the severity of MASH 35-37. Hepatic inflammation is also recognized to perpetuate the progression of hepatic fibrosis 38,39. While HFHC diet-induced mice exhibited increased expression of fibrosis-related proteins, including TGFβ and Col1a1, Ele treatment effectively reversed HFHC-driven inflammatory and fibrotic alterations. Systematic analysis of RNA-seq data revealed that pathways associated with lipid metabolism, inflammation, fibrosis, and apoptosis were enriched and notably suppressed following Ele treatment. Given in the context of MASH* in vivo*, we also validated *in vitro* that Ele significantly increased AMPK phosphorylation, and suppressed inflammation and fibrosis in both mouse and human hepatocytes, respectively. Interestingly, hepatocytes induced by PO exhibited mitochondrial dysfunction, mainly manifesting in the deficiency of mitochondrial respiration and the elevated glycolysis. The current evidence suggested a causal relationship between mitochondrial defect and MASH. Conversely, Ele administration reprogrammed mitochondrial energy metabolic phenotypes, that is, switching from glycolysis inhibition to OXPHOS enhancement, thereby indirectly leading to higher mitochondrial activity.

Has1, which encodes a core enzyme for HA biosynthesis, emerged as the most prominently upregulated transcript within the lipid metabolism pathway in our differential gene expression analysis. It is noteworthy that the upregulation of Has1 observed in our HFHC model contrasts with findings from a CDHFD-induced model 40, underscoring the model-dependent nature of MASH pathogenesis. Our study focused on the HFHC model, which more closely recapitulates the metabolic and fibrotic features of human MASH, but further validation in additional dietary or toxin-induced models will be required to assess the generalizability of these findings. Has1 mRNA was significantly high-expressed in patients with MASH, as evidenced by the GEO database. We acknowledge the limited sample size of our initial human validation cohort, which constrains statistical power. However, subsequent analyses of two independent, large public cohorts consistently demonstrated a significant and substantial upregulation of Has1 expression in MASH livers. This provides strong external validation of our findings and substantially reinforces the credibility of Has1 as a translationally relevant biomarker associated with MASH. A previous report identified that HA is a commonly used indicator in clinical assessment of liver disease progression and serves as a sensitive marker reflecting liver fibrosis, facilitating dynamic monitoring 9,41,42. Analyzing Has1 with genetic deficiency or pharmacological inhibition revealed major changes in the principal pathological phenotypes of MASH. Here we demonstrated that Has1 and HA synthesis level were inhibited in HFHC-fed mice, in mice receiving Ele or 4-MU by oral gavage. The hepatocyte-specific deficiency and pharmacological inhibition of Has1 elicited a remarkable amelioration of hepatic fat deposition, inflammation, fibrosis, and liver function in response to PO-induced hepatocytes and HFHC-fed diet, respectively. Our study revealed the direct effects of Ele within hepatocytes and, through co-culture experiments, elucidated a crucial indirect mechanism, the modulation of hepatocyte-HSC intercellular communication. We demonstrated that hepatocytes under PO stress served as a significant source of pro-fibrotic signals, and targeting Has1 in hepatocytes effectively disrupted these signals, thereby attenuating HSC activation in the *in vitro* models. Although the key paracrine factor derived from hepatocytes remain to be identified in future studies, our findings establish the critical role of the Ele-Has1 pathway in modulating this pathogenic cellular crosstalk. In the present study, we, for the first time, identified that the causal role of Has1 was in the context of MASH *in vivo* and* in vitro*, where the potential effectiveness in humans is underlined on Has1 expression. The negative feedback from lipid-derived metabolites accumulated during mitochondrial FAO underlies the compromised mitochondrial activity in MASH 43. Consequently, Has1 knockdown in hepatocytes maintained mitochondrial respiratory capacity, suggesting that the elevated metabolic flexibility by Has1 suppression facilitated fatty acid oxidation. Our study highlighted that Has1 activation under metabolic stress contributes significantly to metabolic dysregulation observed in MASH. Molecular docking, SPR, CETSA, and co-immunoprecipitation experiments were used to observe the binding mode and real-time kinetics of Ele and Has1, we have essentially concluded that a strong affinity is confirmed between Ele and Has1 protein. Consistent with these findings, both siRNA-mediated AMPK knockdown and pharmacological inhibition with CC further confirmed that AMPK activation is indispensable for Ele-mediated suppression of Has1. Collectively, these results suggest that Ele directly targets Has1 pharmacologically, acting as an inhibitor through binding to Has1 and ameliorating MASH-induced liver injury. While liver-specific Has1 knockdown in HFHC-fed mice would provide definitive genetic validation, our current pharmacological and siRNA-based data consistently indicate that Has1 is a pivotal mediator of the hepatoprotective actions of Ele. Future studies employing hepatocyte-specific Has1 knockdown models will further substantiate this mechanistic link. Given the established role of AMPK in MASH, we further explored its functional connection with Has1 at the molecular level. Remarkably, the interaction between AMPK and Has1 not only exists but also exhibits a negative correlation regulatory relationship, which suggests that targeting the molecular interaction between AMPK and Has1 can selectively lower Has1 activity. It should be noted that this study was restricted to male C57BL/6J mice, which represents a limitation. While this design reduces variability associated with hormonal fluctuations and provides a more consistent MASH phenotype, it does not account for potential sex-specific differences in disease progression. Future studies in female cohorts are essential to confirm that our results generalize.

Metabolic disorders and the accumulation of toxic lipid products are recognized as fundamental contributors to MASH development 44. Hepatic lipid dysregulation remodels the intrahepatic lipid landscape. We therefore systematically compared liver lipidomics profiles from control, HFHC, HFHC+4-MU, and HFHC+Ele groups to identify specific lipids implicated in MASH development and therapeutic response. 4-MU and Ele profoundly altered the total lipid content in the HFHC diet-fed liver, and they both effectively alleviated MASH. We particularly emphasized on the hepatic lipidomic profiles of Ele. Although Ele has been reported as an SCD1 inhibitor, our data clearly distinguished its mechanism from classical SCD1 blockade. MK-8245 treatment and SCD1 overexpression confirmed that the lipidomic profile alterations induced by Ele were not primarily attributable to reduced SCD1 activity, but rather involved the downregulation of Has1 and subsequent activation of AMPK. These results strengthen our conclusion that Ele exerted its anti-MASH effects *via* a novel Has1/AMPK axis independent of SCD1 inhibition. Interestingly, Ele supplementation significantly decreased the overall content of FA, GL, SP, and ST in HFHC-diet mice, while the content of GP markedly increased, especially in PE. Although the relative abundance of PE not only regulates the lipid content in hepatocytes but also influences energy metabolism in mitochondria 45, a lack of comprehensive investigation exists regarding the impact of PE on MASH in murine models. We further aimed to explore the mechanisms through which MASH and Ele modulate PE. Based on *de novo* biosynthesis of PE 46, we summarized the primary changes in biosynthetic and metabolic pathways resulting from Ele administration. PE was further quantified based on the hepatic targeted quantitative lipidomics to more accurately profile the alteration of PE, subsequently indicated that Ele could significantly reverse the decreased PE in the MASH mice. On PO-induced mice hepatocytes, we found that supplement exogenous PE greatly alleviated the protein expression of MASH-like phenotype. As expected, the protein levels of Pisd and Pemt1, critical enzymes in PE biosynthesis and remodeling 4,47,48, were normalized by Ele administration. Although mitochondrial function is impaired in MASH, it remains unclear whether this dysfunction is attributable to PE deficiency. PE supplementation ameliorated homeostasis of mitochondrial energy metabolic phenotype, suggesting enhanced fatty acid oxidation. Our study highlighted the hepatic PE deficiency might contribute to mitochondrial malfunction, which further drove MASH. Therefore, PE may be physiologically significant the pathogenesis and treatment of MASH. A future objective is to investigate how Ele influences PE and related GP enzyme activities, which will clarify its mechanism of action in HFHC-diet mice.

## Conclusions

The current study provides evidence that we systemically elucidate that Ele significantly ameliorated hepatic fat deposition, inflammation, fibrosis, and liver function in response to an HFHC-fed MASH mouse mode and PO-induced hepatocytes. Mechanistically, AMPK activation triggers lipid metabolism reprogramming that alleviates metabolic disorder. Integrative multi-omics analyses combining transcriptomics and lipidomics were used to pinpoint signature genes and lipids involved in MASH development and treatment. The amelioration of MASH following hepatic Has1 deficiency established a valuable platform for elucidating mechanisms driving the progression from steatosis to steatohepatitis and fibrosis. Our findings suggest that Has1 is a direct pharmacological target of Ele to protect against MASH. Interestingly, the interaction between AMPK and Has1 controls hepatic lipid homeostasis and is a hitherto undescribed mechanism of the inhibitory effect of Ele on MASH. More importantly, it exists a close link between PE and MASH, and Ele intervention protected against HFHC diet-triggered liver injury, which was associated with increased levels of PE in the liver. Taken together, our present study provided further evidence that supported the protective effect of Ele against MASH. Collectively, this work adds to the evidence that Ele has therapeutic potential in ameliorating MASH.

## Supplementary Material

Supplementary figures and table.

## Abbreviations

4-MU: 4-Methylumbelliferone
AMPK: AMP-activated protein kinase
AST: Aspartate transaminase
DNL: *De novo* lipogenesis
BSA: Bovine serum albumin
ACC: Acetyl-CoA carboxylase
BUN: Blood urea nitrogen
Compound C: CC
CPT-1A: Carnitine palmitoyltransferase 1α
Cr: Creatinine
ECAR: Extracellular acidification rate
Ele: Elemicin
CETSA: Cellular thermal shift assay
FASN: Fatty acid synthase
HA: Hyaluronan
Has1: Hyaluronan synthase 1
FAO: Fatty acid oxidation
HFHC: High-fat/high-cholesterol
IL-18: Interleukin-18
ITC: Isothermal titration calorimetry
IL-1β: Interleukin-1β
LDL-C: Low-density lipoprotein cholesterol
MASLD: Metabolic dysfunction-associated steatotic liver disease
NAFLD: Nonalcoholic fatty liver disease
NAS: NAFLD activity score
MASH: Metabolic dysfunction-associated steatohepatitis
NLRP3: NOD-like receptor protein 3
ASC: Apoptosis-associated speck-like protein containing a CARD
OCR: Oxygen consumption rate
SREBP-1c: Sterol regulatory element binding protein-1c
MASL: Metabolic-associated steatotic liver
ORO: Oil Red O
SCD1: Stearoyl-CoA desaturase 1
PA: Phosphatidate
PC: Phosphatidylcholine
PE: Phosphatidylethanolamine
PI: Phosphatidylinositols
PO: Palmitic acid/oleic acid
PPARα: Peroxisome proliferator-activated receptor α
TC: Total cholesterol
PS: Phosphatidylserines
TGFβ: Transforming growth factor β
TG: Triglyceride
