# Supplementary material for: Therapeutic targeting of the AMPK-Has1 complex formation ameliorates metabolic dysfunction-associated steatohepatitis in mice
Source: Theranostics. 2026 Jan 22;16(8):4260–82. doi: 10.7150/thno.120527 (PMC12905822; doi:10.7150/thno.120527)
Supplement: Supplementary file 1 — Supplementary figures and table. [file thnov16p4260s1.pdf]

Therapeutic targeting of the AMPK-Has1 complex formation ameliorates metabolic dysfunction-associated steatohepatitis in mice

Xunzhe Yin[1,2], Wenjing Zhao[4], Li Yang[4], Chang Li[2], Xiangyu Guo[2], Lihao Lin[2], Zuojia Liu[2,*], Jin Wang[1,3,*]

[1] Center for Theoretical Interdisciplinary Sciences, Wenzhou Institute, University of Chinese Academy of Sciences, Wenzhou, Zhejiang 325001, China.

[2] State Key Laboratory of Electroanalytical Chemistry, Changchun Institute of Applied Chemistry, Chinese Academy of Sciences, Changchun, Jilin 130022, China.

[3] Department of Chemistry and of Physics and Astronomy, Stony Brook University, Stony Brook, NY 11794-3400, USA.

[4] Hepatobiliary Hospital of Jilin Province, Changchun, Jilin 130062, China.

[*]Corresponding authors.

Professor Zuojia Liu at State Key Laboratory of Electroanalytical Chemistry, Changchun Institute of Applied Chemistry, Chinese Academy of Sciences, No. 5625, Ren Min Street, Changchun 130022, Jilin, China. Email: zjliu@ciac.ac.cn (Z. Liu).

Professor Jin Wang at Center for Theoretical Interdisciplinary Sciences, Wenzhou Institute, University of Chinese Academy of Sciences, Wenzhou, Zhejiang 325001, China. Department of Chemistry and of Physics and Astronomy, Stony Brook University, Stony Brook, New York 11794-3400, USA. Email: jin.wang.1@stonybrook.edu (J. Wang).

Supplementary tables

Table S1. Primer sequences for qPCR.

**Gene**	**Sequence (5'-3')**	
Mouse *Fasn*	F:	CTGCGGAAACTTCAGGAAATG
	R:	GGTTCGGAATGCTATCCAGG
Mouse *Cpt1α*	F:	TGGCATCATCACTGGTGTGTT
	R:	GTCTAGGGTCCGATTGATCTTTG
Mouse *Il1b*	F:	CCGTGGACCTTCCAGGATGA
	R:	GGGAACGTCACACACCAGCA
Mouse *Ccl5*	F:	TGCTGCTTTGCCTACCTCTC
	R:	TCTTCTCTGGGTTGGCACAC
Mouse *Srebp*	F:	GTGAGCCTGACAAGCAATCA
	R:	GGTGCCTACAGAGCAAGAG
Mouse *Hmgcr*	F:	TGTTCACCGGCAACAACAAGA
	R:	CCGCGTTATCGTCAGGATGA
Mouse *Acc*	F:	GGCCAGTGCTATGCTGAGAT
	R:	AGGGTCAAGTGCTGCTCCA
Mouse *Pparα*	F:	GAGCTGCAAGATTCAGAAGAAG
	R:	GAATCTTTCAGGTCGTGTTCAC
Mouse *Tnfα*	F:	CATCTTCTCAAAATTCGAGTGACAA
	R:	TGGGAGTAGACAAGGTACAACCC
Mouse *Il6*	F:	TAGTCCTTCCTACCCCAATTTCC
	R:	TTGGTCCTTAGCCACTCCTTC
Mouse *Ccl2*	F:	TACAAGAGGATCACCAGCAGC
	R:	ACCTTAGGGCAGATGCAGTT

Mouse *Cxcl10*	F:	ATGACGGGCCAGTGAGAATG
	R:	ATGATCTCAACACGTGGGCA
Mouse *Has1*	F:	TATGCTACCAAGTATACCTCG
	R:	TCTCGGAAGTAAGATTTGGAC
Mouse *β-actin*	F:	GTGACGTTGACATCCGTAAAGA
	F:	GCCGGACTCATCGTACTCC

Supplementary figures

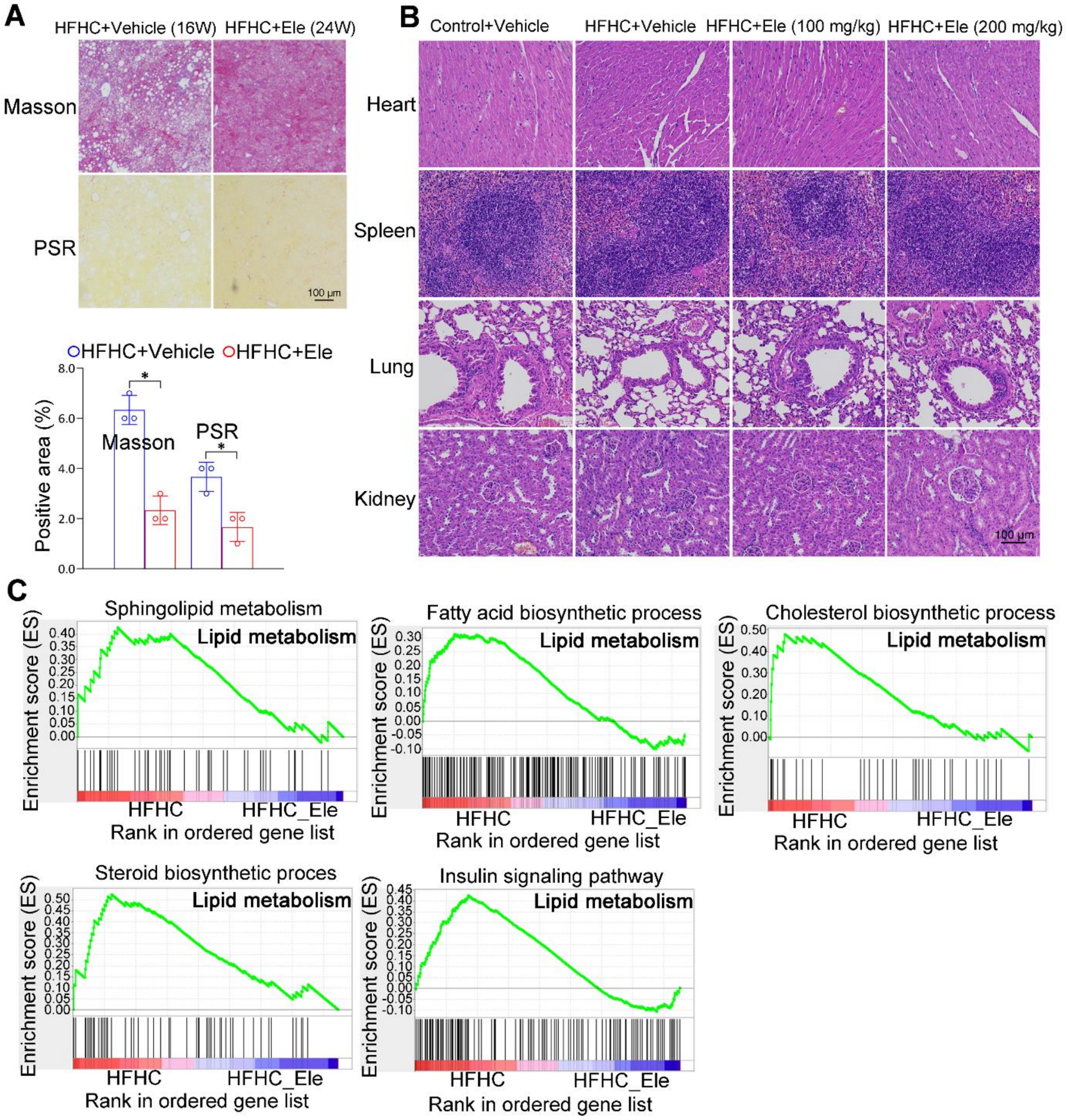

Figure S1. (A) Representative Masson and PSR staining of liver sections from mice treated with vehicle or Ele, collected at 16 weeks of HFHC feeding (initiation) and at the conclusion of the treatment phase, showing fibrosis levels (mean ± SEM, $n = 6$). (Scale bars, 100 μm). (B) Representative images of H&E staining of HFHC diet-induced mice heart, spleen lung, and kidney section treated with vehicle or indicated doses of Ele. (Scale bars, 100 μm). (C) Significant enrichment of lipid metabolism-related pathways in Ele-treated mouse liver, including s sphingolipid metabolism, fatty acid biosynthetic process, cholesterol biosynthetic

process, steroid biosynthetic process, and insulin signaling pathway, as revealed by GSEA analysis of RNA-seq data versus vehicle controls. ($^{*}P < 0.05$, $^{**}P < 0.01$ *vs.* HFHC+Vehicle).

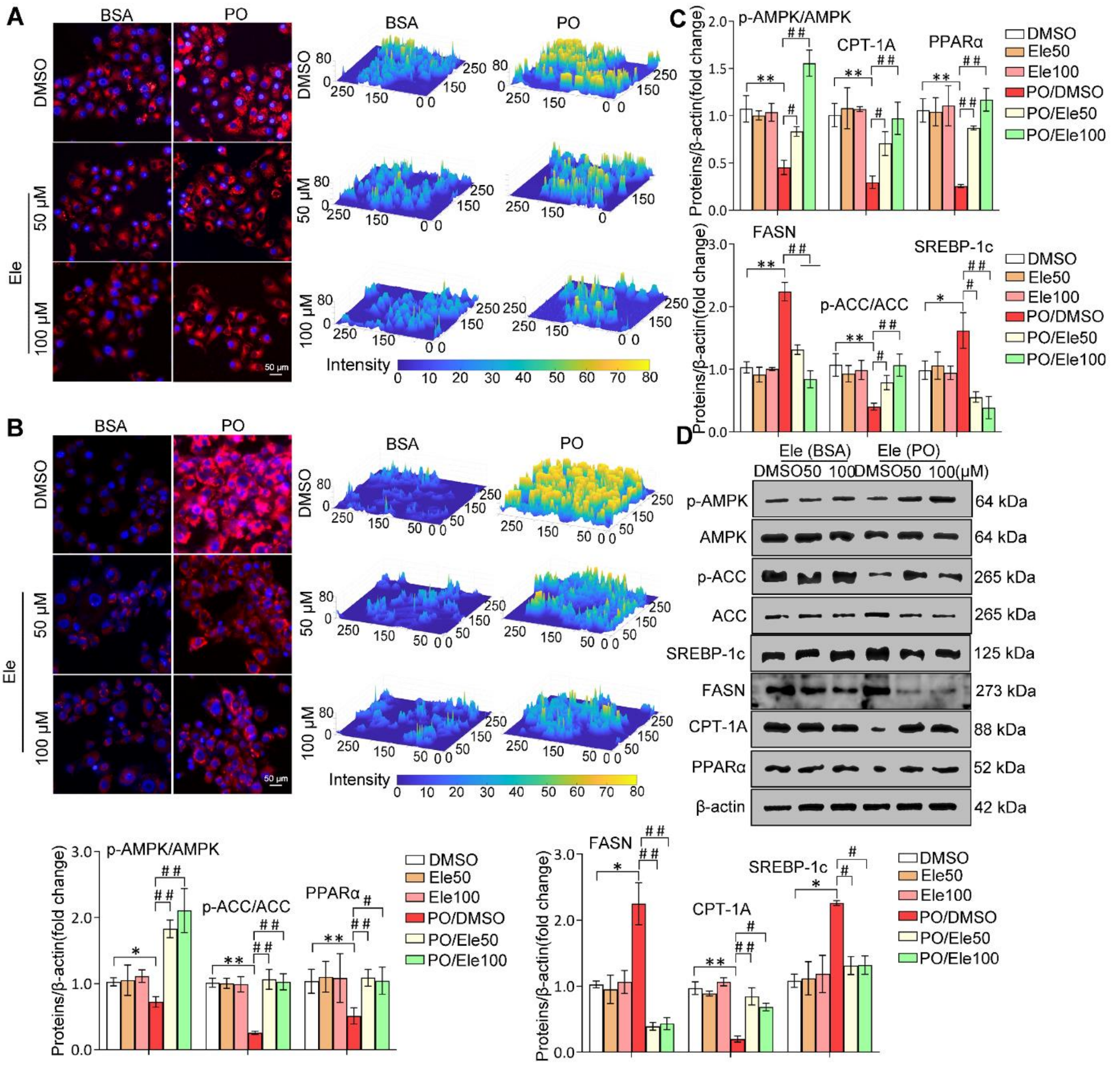

Figure S2. Nile Red staining and quantifications of mouse primary hepatocytes (A) and human L02 hepatocytes (B) administered with DMSO and Ele (50 and 100 μM) in response to BSA or PO stimulation for 24 h. (mean ± SEM, *n* = 5). (Scale bar, 100 μm.) (C) Relative quantifications showing AMPK, p-AMPK, ACC, p-ACC, SREBP-1c, FASN, CPT-1A, and PPARα proteins in primary hepatocytes in response to the corresponding stimulus (mean ± SEM, *n* = 3). (D) Western blot analysis and quantifications of AMPK, p-AMPK, ACC, p-ACC, SREBP-1c, FASN, CPT-1A, and PPARα proteins in human L02 hepatocytes in response to the corresponding stimulus (mean ± SEM, *n* = 3). ($^{*}P < 0.05$, $^{**}P < 0.01$ *vs.* DMSO; $^{\#}P < 0.05$, $^{\#\#}P < 0.01$ *vs.* PO+DMSO).

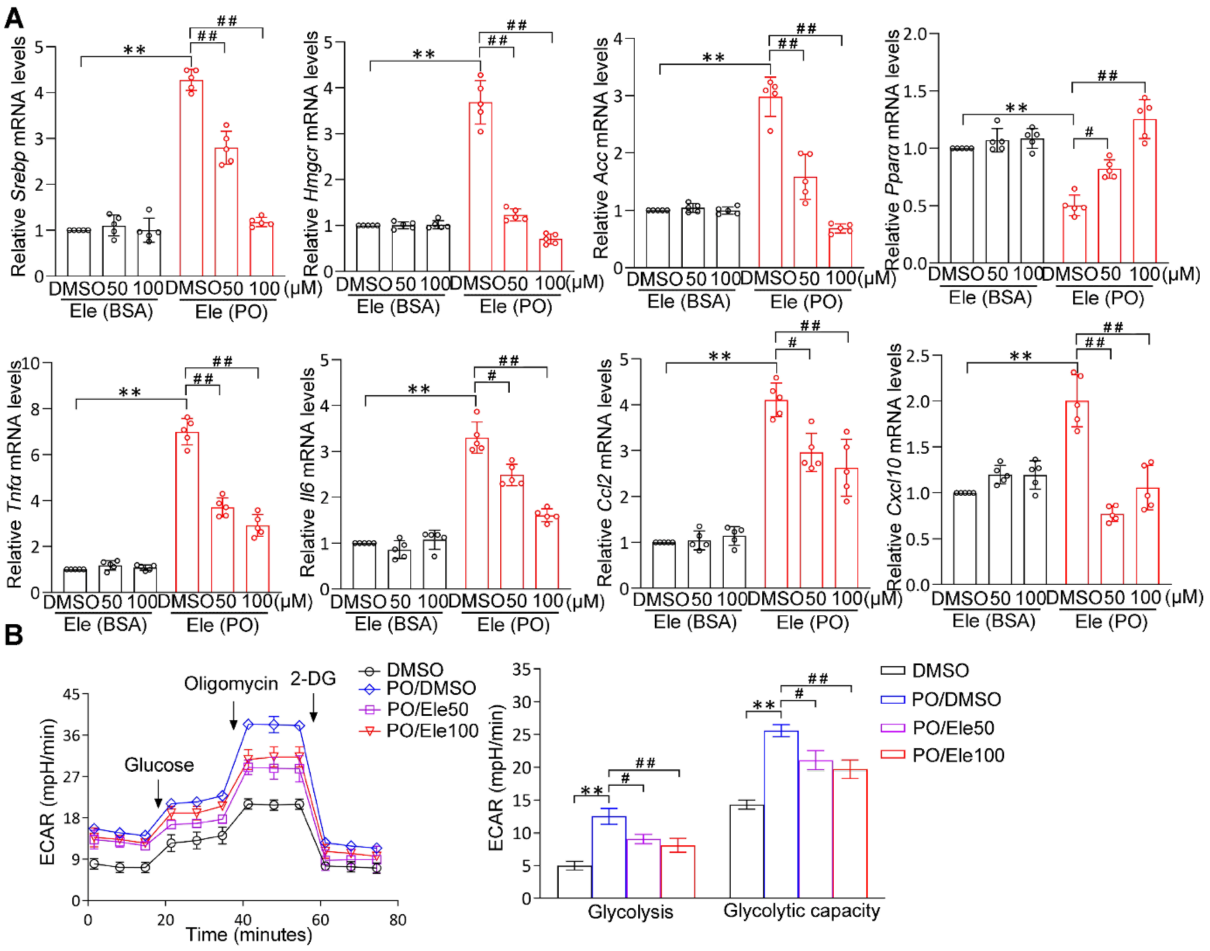

Figure S3. (A) The relative mRNA levels of *Srebp*, *Hmgcr*, *Acc*, *Pparα*, *Tnfα*, *Il6*, *Ccl2*, and *Cxcl10* genes were determined by real-time PCR in primary hepatocytes in response to the corresponding stimulus, and normalized using *β-actin* as an internal control (mean ± SEM, n = 5). (B) Representative and parametric results of ECAR in primary hepatocytes treated with DMSO or Ele (mean ± SEM, n = 3). Oligomycin, ATP synthase inhibitor; 2-DG, competitive inhibitor of glucose ($^{*}P < 0.05$, $^{**}P < 0.01$ *vs.* DMSO; $^{\#}P < 0.05$, $^{\#\#}P < 0.01$ *vs.* PO+DMSO).

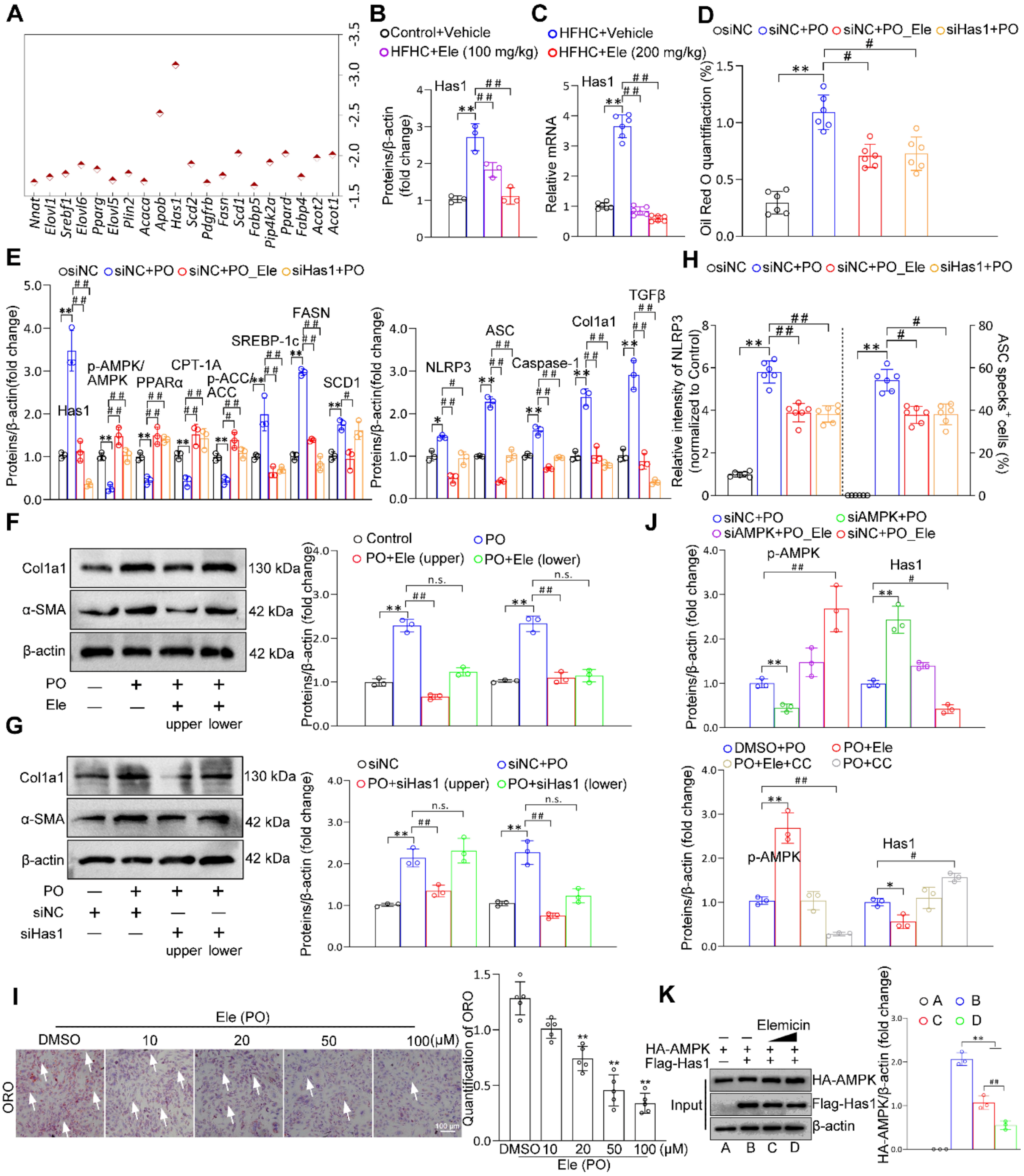

Figure S4. (A) The screening criteria prioritized genes based on log_2FoldChange magnitude. The quantifications of western blot (B) and mRNA level (C) of Has1 in livers from mice treated with vehicle or indicated doses of Ele (mean ± SEM, *n* = 3 in western blot and *n* = 5 in qPCR). (D) The quantification of oil red O staining in primary hepatocytes in response to PO stimulation treated with/without Ele or siHas1 (mean ± SEM, *n* = 6). (E) The corresponding quantifications of protein expression, including Has1, p-AMPK, AMPK, PPARα, CPT-1A, p-

ACC, ACC, SREBP-1c, FASN, SCD1, NLRP3, ASC, Caspase-1, Col1a1, and TGFβ proteins in PO-induced primary hepatocytes under the indicated conditions (mean ± SEM, n = 3). (F) Representative western blot images showing Col1a1 and α-SMA protein expression in response to the respective stimuli in a non-contact co-culture system of L02 and LX-2 cells (mean ± SEM, n = 3). (G) Representative western blot images showing Col1a1 and α-SMA protein expression in response to Has1 siRNA transfection in a non-contact co-culture system of L02 and LX-2 cells (mean ± SEM, n = 3). (H) The quantifications of immunofluorescence staining of ASC and NLRP3 in PO-induced primary hepatocytes with indicated treatments. (I) ORO staining and quantifications of lipid accumulation in hepatocytes treated with varying concentrations of Ele in response to PO stimulation. (mean ± SEM, n = 5). (Scale bar, 100 μm.) (J) Quantification of p-AMPK and Has1 protein levels in hepatocytes stimulated with PO and treated with Ele, siAMPK, or CC (mean ± SEM, n = 3). (K) 293T cells were co-transfected with Flag-labeled Has1, and HA-labeled AMPK. Cell lysates were immunoprecipitated with immunomagnetic beads and immunoblotted with the indicated antibodies (mean ± SEM, n = 3). ($^{*}P < 0.05$, $^{**}P < 0.01$; $^{\#}P < 0.05$, $^{\#\#}P < 0.01$).

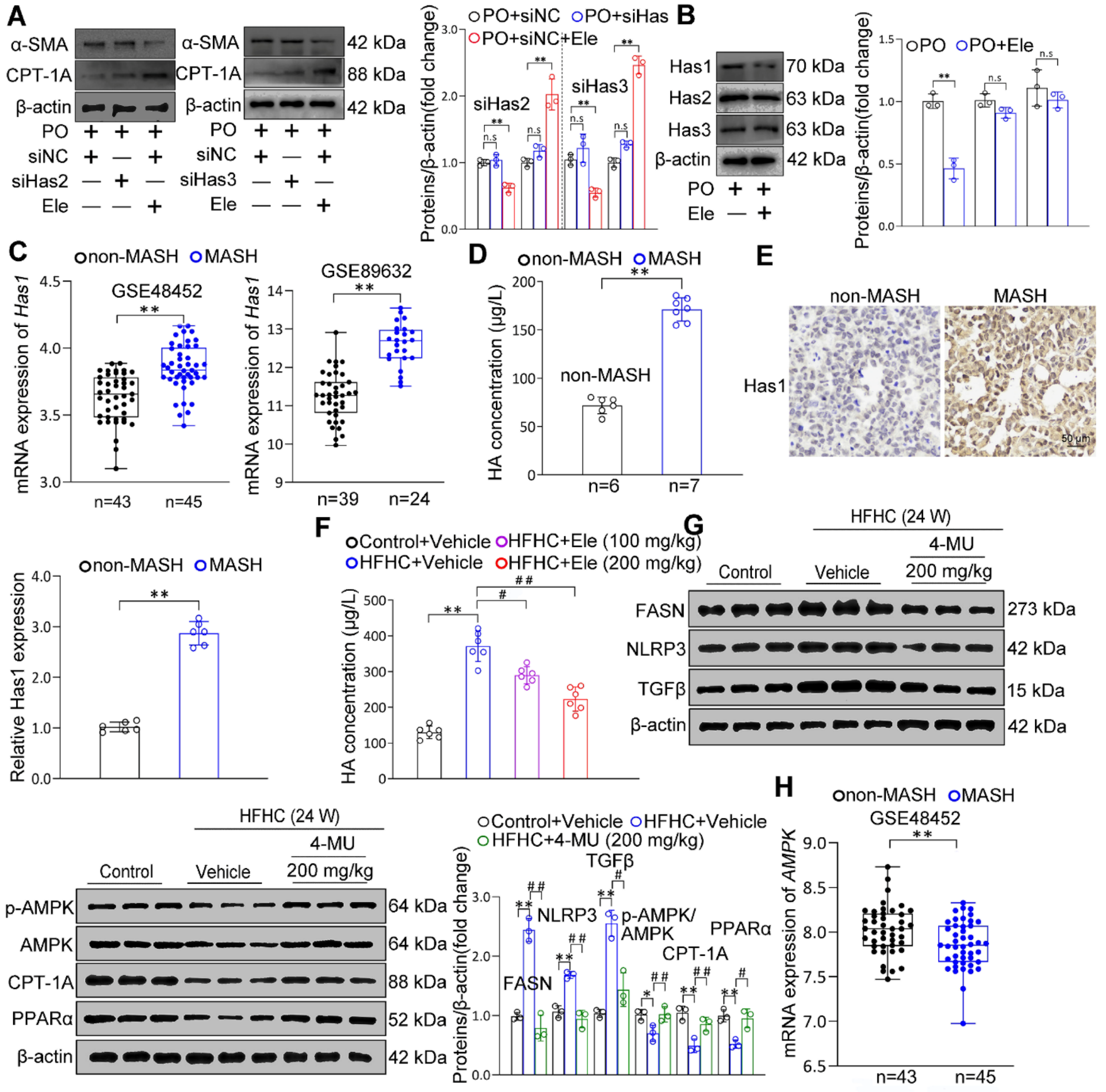

Figure S5. (A) Western blot analysis and quantifications of α-SMA and CPT-1A proteins in hepatocytes treated with Has2 or Has3 siRNA (mean ± SEM, $n = 3$). (B) Western blot analysis and quantifications of Has1, Has2, and Has3 proteins in hepatocytes in response to the corresponding stimulus (mean ± SEM, $n = 3$). (C) The expression levels of Has1 gene in MASH and matched normal liver tissues based on GEO GSE48452 (mean ± SEM, $n = 43$ in non-MASH and $n = 45$ in MASH) and GSE89632 (mean ± SEM, $n = 39$ in non-MASH and $n = 24$ in MASH). (D) Serum hyaluronan content detection of clinical human and corresponding normal serum (mean ± SEM, $n = 6$ in non-MASH and $n = 7$ in MASH). (E) Representative IHC staining and quantification of Has1 in liver sections from patients with non-MASH and MASH

(mean ± SEM, n = 6 in non-MASH and n = 7 in MASH). (Scale bar, 50 μm.) (F) Serum hyaluronan content detection of HFHC diet-induced mice treated with Ele (mean ± SEM, n = 6). (G) Western blot analysis and quantifications of p-AMPK, AMPK, PPARα, CPT-1A, FASN, NLRP3, and TGFβ proteins in livers of mice treated with 4-MU or vehicle (mean ± SEM, n = 3). (H) The expression levels of AMPK gene based on GEO GSE48452 (mean ± SEM, n = 43 in non-MASH and n = 45 in MASH). ($^{*}P < 0.05$, $^{**}P < 0.01$; $^{\#}P < 0.05$, $^{\#\#}P < 0.01$).

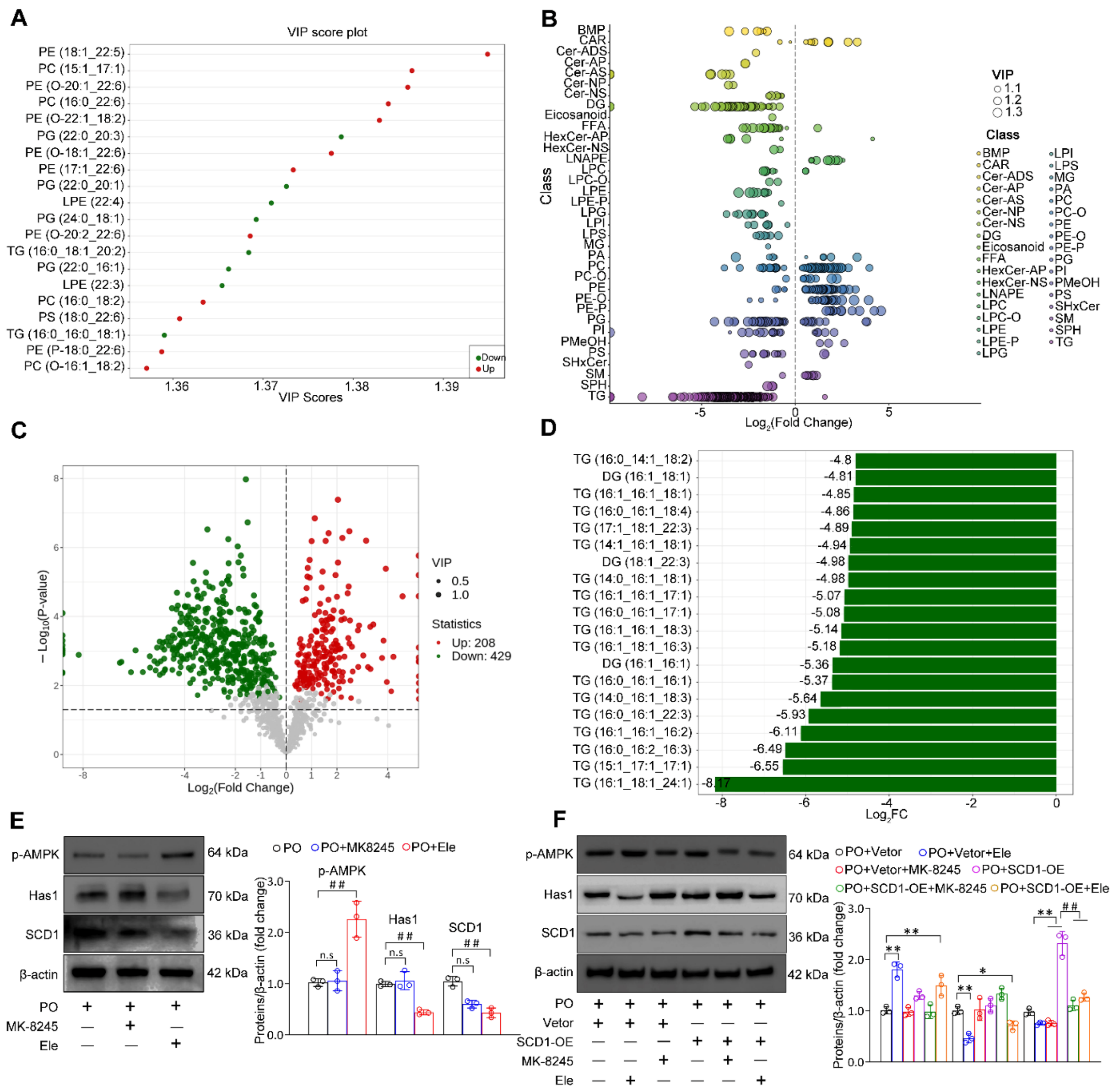

Figure S6. (A) Comparison and summary in hepatic lipidomics of differentially altered lipid metabolites (DALs) identified based on screening criteria across the HFHC and Ele groups, with final display of top 20 DALs showing highest VIP values in OPLS-DA model. (B) Scatter plot visualization of DALs depicting content variations of diverse lipid subclasses under the indicated conditions, showing content differences for each lipid and DALs across comparison groups. (C) Volcano Plot visualization displaying lipid content differences between HFHC and Ele groups and statistical significance of these differences. (D) Qualitative and quantitative analysis of lipids in HFHC and Ele groups, highlighting top 20 downregulated lipids with largest quantitative differences. (E, F) Western blot analysis and quantifications of p-AMPK,

Has1, and SCD1 proteins in hepatocytes in response to the corresponding stimulus (mean ± SEM, $n = 3$). ($^{*}P < 0.05$, $^{**}P < 0.01$; $^{\#}P < 0.05$, $^{\#\#}P < 0.01$).

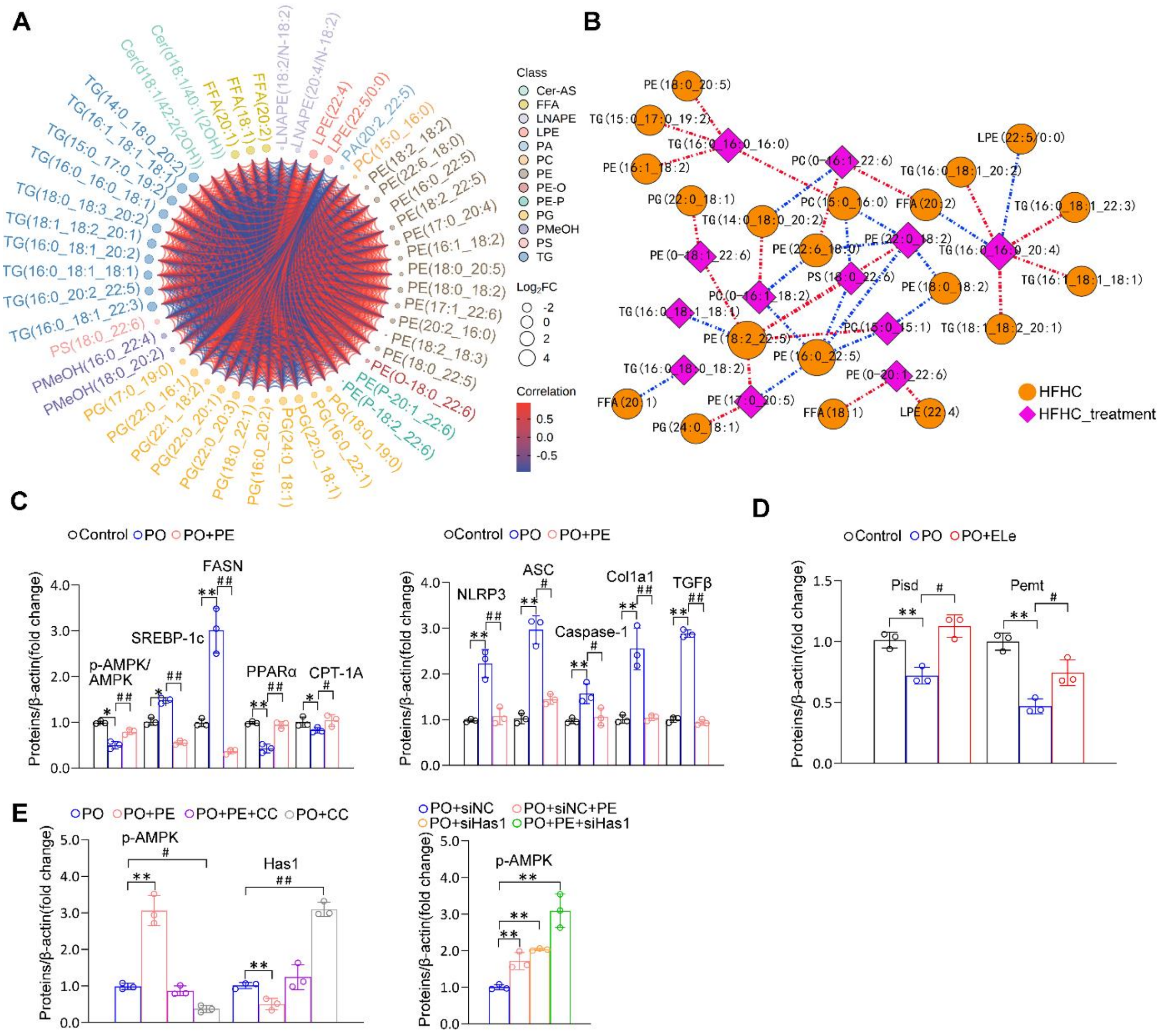

Figure S7. (A, B) Correlation analysis of hepatic lipidomics to display synergistic or antagonistic relationships among different lipids and to assess metabolic proximities of DALs between HFHC-induced livers treated with vehicle or Ele. Dot size represents the magnitude of the log_2FC value (larger dots indicate greater log_2FC values), dot color denotes the lipid subclass, and connecting lines indicate correlation coefficients between lipid species, with darker colors reflecting higher absolute values. (C, D) The corresponding quantifications of protein expression, including p-AMPK, SREBP-1c, FASN, PPARα, CPT-1A, NLRP3, ASC, Caspase-1, Col1a1, TGFβ, Pisd, and Pemt proteins in primary hepatocytes in response to PO stimulation treated with PE (mean ± SEM, n = 3). (E) Quantification of p-AMPK or Has1 protein levels in hepatocytes stimulated with PO and treated with PE, CC, or siHas1 (mean ± SEM, n = 3). ($^{*}P < 0.05$, $^{**}P < 0.01$; $^{\#}P < 0.05$, $^{\#\#}P < 0.01$).
